# Multiscale insights into screw dislocation-driven growth mechanisms

**DOI:** 10.1126/sciadv.aei7996

**Published:** 2026-10-09

**Authors:** Peiyu Qiao, Zhongshi Zhang, Bohui Xu, Pifu Gong, Xuanze Li, Yifan Wang, Lifeng Tian, Pei Liu, Jianyu Cao, Yuye Li, Qi Zhang, Chun Zhao, Xiangmin Meng

**Affiliations:** ^1^ Key Laboratory of Photochemical Conversion and Optoelectronic Materials, Technical Institute of Physics and Chemistry, Chinese Academy of Sciences, Beijing, 100190, China.; ^2^ School of Advanced Technology, Xi'an Jiaotong-Liverpool University, Suzhou, 215123, China.; ^3^ Functional Crystals Lab, Key Laboratory of Functional Crystals and Laser Technology, Technical Institute of Physics and Chemistry, Chinese Academy of Science, Beijing, 100190, PR China.; ^4^ Institute of Process Engineering, Chinese Academy of Sciences, Beijing, 100190, China.; ^5^ Interior &amp; Exterior Trim Department 1, Beijing Automotive Research Institute Co. Ltd., Beijing, 100021, China.; ^6^ State Key Lab of Metal Matrix Composites, School of Materials Science and Engineering, Shanghai Jiao Tong University, Shanghai, 200240, P.R. China.

## Abstract

Spiral growth in two-dimensional (2D) materials has conventionally been explained by single screw dislocation mechanisms, yet a complete multiscale understanding remains elusive due to the intricate nature of screw dislocation structures. Here, we present a stabilization approach through metallic transition metal dichalcogenides (m-TMDs) overlayers on spiral semiconducting TMDs (s-TMDs), which enables unprecedentedly robust atomic-resolution imaging of screw dislocations in cross-sectional van der Waals heterostructures. Our multiscale investigations identify nanoscale substrate undulations as critical nucleation sites for screw dislocations and uncover a multi-screw dislocation-driven (MSDD) growth paradigm, characterized by periodic stress stripes with linear densities approaching ∼10^6^ cm^−1^. Furthermore, we establish that dislocation interactions govern stacking sequence reconstruction, while the kinetic competition among multiple screw dislocations determines the ultimate spiral architectures. These insights fundamentally transform our understanding of defect-mediated growth in 2D materials.

## INTRODUCTION

Understanding the mechanisms of crystal growth is essential for the bottom-up synthesis of nanomaterials with tunable sizes and morphologies. According to the classical Burton-Cabrera-Frank (BCF) theory (*1*–*4*), screw dislocations provide a self-perpetuating growth step that eliminates the need for catalysts or templates, breaking the symmetry of crystal growth and resolving the paradox of rapid growth under low supersaturation conditions. Specifically, an axial screw dislocation in a one-dimensional (1D) nanowire material can induce continuous lattice twisting, known as the Eshelby twist (*5*, *6*), leading to the formation of helical nanowires or even complex nanowire trees (*7*–*10*). Similarly, screw dislocation-driven (SDD) growth is widely observed in two-dimensional (2D) van der Waals (vdW) layered materials, such as graphene and transition metal dichalcogenides (TMDs) (*11*–*14*). The tunable interlayer spacing of these materials positions them as ideal platforms for investigating screw dislocations. Recent theoretical and experimental studies have shown that multiple screw dislocations may exist within the spiral structures of 2D materials (*15*, *16*). It is particularly noteworthy that substrates used for material growth are rarely atomically flat and typically feature nanoscale undulations, such as bumps, pleats, grooves, and steps (*17*–*20*). These topological structures provide a natural template for the nucleation and expansion of multiple screw dislocations. However, the interaction between atomic-scale screw dislocations and these curved substrate topographies in promoting macroscopic spiral growth remains poorly understood. Addressing this gap necessitates the development of advanced multiscale characterization methods to elucidate the fundamental mechanisms driving spiral growth.

With the advancement of aberration-corrected electron microscopy, spatial resolution has reached sub-angstrom levels, offering unprecedented opportunities to image defects at the atomic scale (*21*–*24*). In the normal plane of an edge dislocation, atomic columns are subjected to tensile or compressive strain, allowing for the detailed characterization of edge dislocations along the dislocation line in an end-on view with atomic resolution. In contrast, for screw dislocations, the atomic columns parallel to the dislocation line are displaced along the screw axis, forming a helicoid structure that prevents an end-on view, as the displacement occurs parallel to the observation direction. Screw dislocations can be studied through cross-sectional samples prepared using the focused ion beam (FIB) technique. Due to their ultrathin structure, 2D materials exhibit high sensitivity to high-energy ion beams, rendering them prone to amorphization under such conditions (*25*–*28*). Therefore, accurate characterization of screw dislocations in cross-sectional samples is crucial for advancing the understanding of SDD growth mechanisms.

2D TMDs, including 2D semiconducting TMDs (s-TMDs) such as MoX_2_ and WX_2_ (where X = S, Se, Te), as well as their metallic counterparts (m-TMDs) such as VX_2_, NbX_2,_ and TaX_2_, have been extensively studied due to their fascinating properties, including ultrathin layered structure, thickness-dependent bandgap, and valley polarization (*29*–*32*). To date, spiral growth has generally been observed in s-TMDs, while controlled growth of m-TMDs with fewer layers or monolayers remains challenging (*12*–*14*, *33*–*36*).

In this work, we present a multiscale study of SDD growth mechanisms through the synthesis of van der Waals heterostructures (vdWHs) composed of m-TMDs grown on spiral s-TMDs templates, including TaSe_2_/Spiral WS_2_, TaSe_2_/Spiral WSe_2_, TaSe_2_/Spiral MoS_2_, NbSe_2_/Spiral WS_2_, and NbSe_2_/Spiral MoS_2_. The epitaxial growth of m-TMDs effectively preserves the microstructure of s-TMDs during FIB preparation of cross-sectional samples. Using scanning transmission electron microscopy (STEM), we reveal the critical role of nanoscale undulations on the substrate in promoting the formation of screw dislocations. More importantly, at the mesoscopic scale, we identify a multi-screw dislocation-driven (MSDD) growth mechanism through the stress stripes generated by stacking, with an estimated screw dislocation linear density of 10^6^ cm^−1^. Additionally, interactions among adjacent screw dislocations induce interlayer stacking reconstruction, leading to the formation of new stacking phases. Finally, we demonstrate that the kinetic competition among multiple screw dislocations governs the macroscopic morphology.

## RESULTS

### Synthesis and characterization of m-TMDs/Spiral s-TMDs vdWHs

The fabrication and growth processes of m-TMDs/Spiral s-TMDs vdWHs are schematically illustrated in Fig. 1, A and B, respectively. Initially, a modified chemical vapor deposition (CVD) method (fig. S1A) was employed, utilizing tilted SiO_2_/Si substrates to prepare 2D spirally grown s-TMDs, such as WS_2_ and MoS_2_, which exhibited hexagonal and triangular morphologies (Fig. 1, C and D, and figs. S2 and S3). These Spiral s-TMDs templates were subsequently used for the vdW epitaxial growth of 2D m-TMDs, including TaSe_2_ and NbSe_2_, resulting in the formation of m-TMDs/Spiral s-TMDs vdWHs (Fig. 1, E and F, and fig. S1B). Consistent with previous studies (*37*, *38*), multilayer m-TMDs preferentially nucleate at multiple sites along both the edges and spiral edges of the s-TMDs, and progressively cover the entire 2D material surface (fig. S4). The uniformity of the epitaxially grown m-TMDs was confirmed through scanning electron microscopy (SEM) and atomic force microscopy (AFM) analyses (Fig. 1, G to J).

**Fig. 1.**
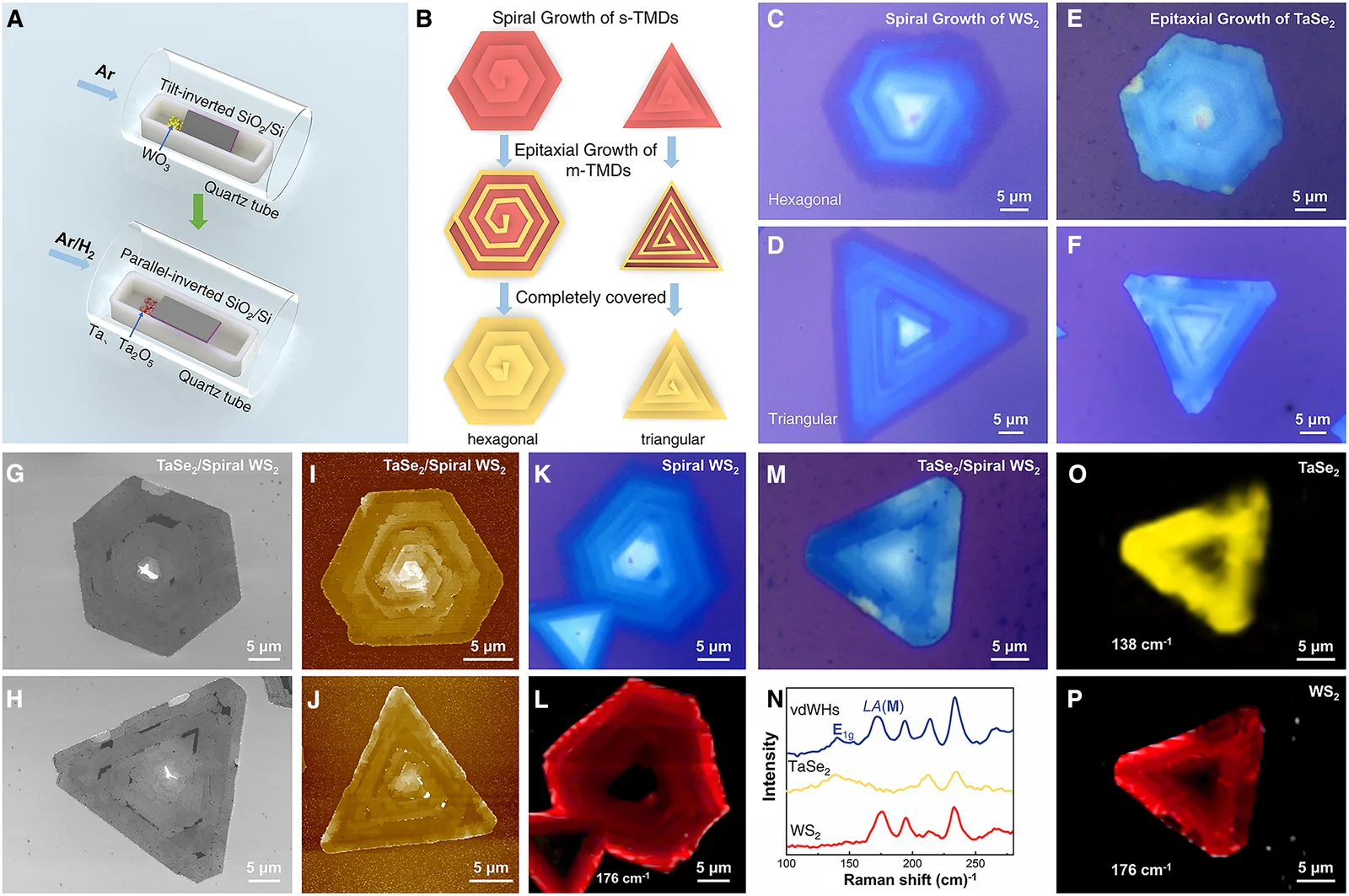
Synthesis and characterization of TaSe_2_/Spiral WS_2_ vdWHs. (**A**) Schematic diagram of the two-step CVD process for synthesizing m-TMDs/Spiral s-TMDs vdWHs. Created with Blender. (**B**) Schematic illustrating the epitaxial growth of m-TMDs on Spiral s-TMDs with hexagonal and triangular morphologies. (**C** and **D**) Optical microscopy images of a hexagonal (C) and a triangular (D) spirally grown 2D WS_2_. (**E** and **F**) Optical microscopy images of a hexagonal (E) and a triangular (F) 2D TaSe_2_/Spiral WS_2_ vdWH, respectively. (**G** and **H**) SEM images of a hexagonal (G) and a triangular (H) 2D TaSe_2_/Spiral WS_2_ vdWH, respectively. (**I** and **J**) AFM images of two representative 2D TaSe_2_/Spiral WS_2_ vdWHs. (**K** and **L**) Optical image (K) and Raman mapping image (L) of two spirally grown 2D WS_2_ samples. (**M** to **P**) Optical image (M), Raman spectra (N), and Raman mapping images (O and P) of a 2D TaSe_2_/Spiral WS_2_ vdWH.

The structural and optical properties of the spirally grown samples were characterized using Raman spectroscopy. For the spirally grown WS_2_ (Fig. 1K), the Raman spectra (Fig. 1N) displayed characteristic peaks consistent with the literature (*39*). Notably, the Raman mapping of the spirally grown WS_2_ (Fig. 1L) revealed a distinct contrast between the central region and the edges, with the central region appearing darker. This contrast is associated with the longitudinal acoustic mode at the M point in the Brillouin zone, *LA*(**M**) (∼176 cm^−1^), a mode highly sensitive to defects (*39*, *40*). The reduced intensity of the *LA*(**M**) mode in the central region indicates a lower defect density on the surface of the spirally grown WS_2_ in this area. Further Raman characterization of the m-TMDs/Spiral s-TMDs vdWHs identified two characteristic peaks in the Raman spectra of the heterostructure samples (Fig. 1, M and N), corresponding to the *LA*(**M**) mode of WS_2_ and the *E*_1g_ mode of TaSe_2_, respectively (*39*, *41*). Raman mapping images further validated the formation of vertically stacked m-TMDs/Spiral s-TMDs vdWHs (Fig. 1, O and P, and fig. S5). Similar to the spirally grown WS_2_, the Raman mapping of the m-TMDs/Spiral s-TMDs vdWHs showed that the central region was darker than the edges. In the case of TaSe_2_, this phenomenon can be attributed to the attenuation of the *E*_1g_ mode intensity with increasing thickness (*41*).

Using this approach, a variety of m-TMDs/Spiral s-TMDs vdWHs, including TaSe_2_/Spiral WSe_2_, TaSe_2_/Spiral MoS_2_, NbSe_2_/Spiral WS_2_, and NbSe_2_/Spiral MoS_2,_ were successfully synthesized (fig. S6) (*39*, *41*–*44*). Optical microscopy and Raman spectroscopy confirmed that all these heterostructures exhibit spiral epitaxial growth. Under the experimental conditions, the epitaxial growth of TaSe_2_ typically resulted in complete coverage of the underlying Spiral s-TMDs, whereas NbSe_2_ epitaxy failed to achieve full coverage of the Spiral s-TMDs. This discrepancy likely arises from variations in the in-plane growth rates of the m-TMDs (figs. S6 and S7).

### Planar-view structural analysis of m-TMDs/Spiral s-TMDs vdWHs

The atomic structure of m-TMDs/Spiral s-TMDs vdWHs was investigated using planar-view high-angle annular dark-field scanning transmission electron microscopy (HAADF-STEM) (Fig. 2A). 2D NbSe_2_/Spiral WS_2_ vdWHs were selected for this study due to the distinctive incomplete epitaxial growth of NbSe_2_ (fig. S7). Energy-dispersive x-ray spectroscopy (EDS) maps (Fig. 2B) of the white-dashed region in Fig. 2A reveal a spiral laminar distribution of W and S elements in the underlying WS_2_. Simultaneously, Nb and Se elements exhibit a laminar epitaxial structure with interlayer gaps, consistent with the observed surface morphology. Selected area electron diffraction (SAED) patterns display a single set of hexagonally arranged diffraction spots (Fig. 2C) in the bare WS_2_ region (red dot in Fig. 2A) and two sets of identically oriented diffraction spots (Fig. 2D) in the overlapping NbSe_2_/Spiral WS_2_ region (yellow dot in Fig. 2A). The inner set corresponds to NbSe_2_, while the outer set is attributed to WS_2_, aligning with their respective lattice constants (∼0.34 nm for NbSe_2_ and ∼0.32 nm for WS_2_). The HAADF-STEM image shows a distinct moiré superlattice formed between the similarly oriented NbSe_2_ and WS_2_ lattices (Fig. 2E), with a periodic unit of 3.79 ± 0.01 nm, equivalent to 11 × 11 NbSe_2_ unit cells stacked on 12 × 12 WS_2_ unit cells, consistent with the theoretical model (Fig. 2F). A high-magnification image of the interface (Fig. 2G) illustrates the moiré superlattice in the upper half and the WS_2_ lattice in the lower half, while an enlarged view (Fig. 2H) further confirms the WS_2_ lattice structure (fig. S8). These findings highlight the well-defined chemical distributions and atomic structures of m-TMDs/Spiral s-TMDs vdWHs, offering valuable insights into the mechanisms underlying spiral growth.

**Fig. 2.**
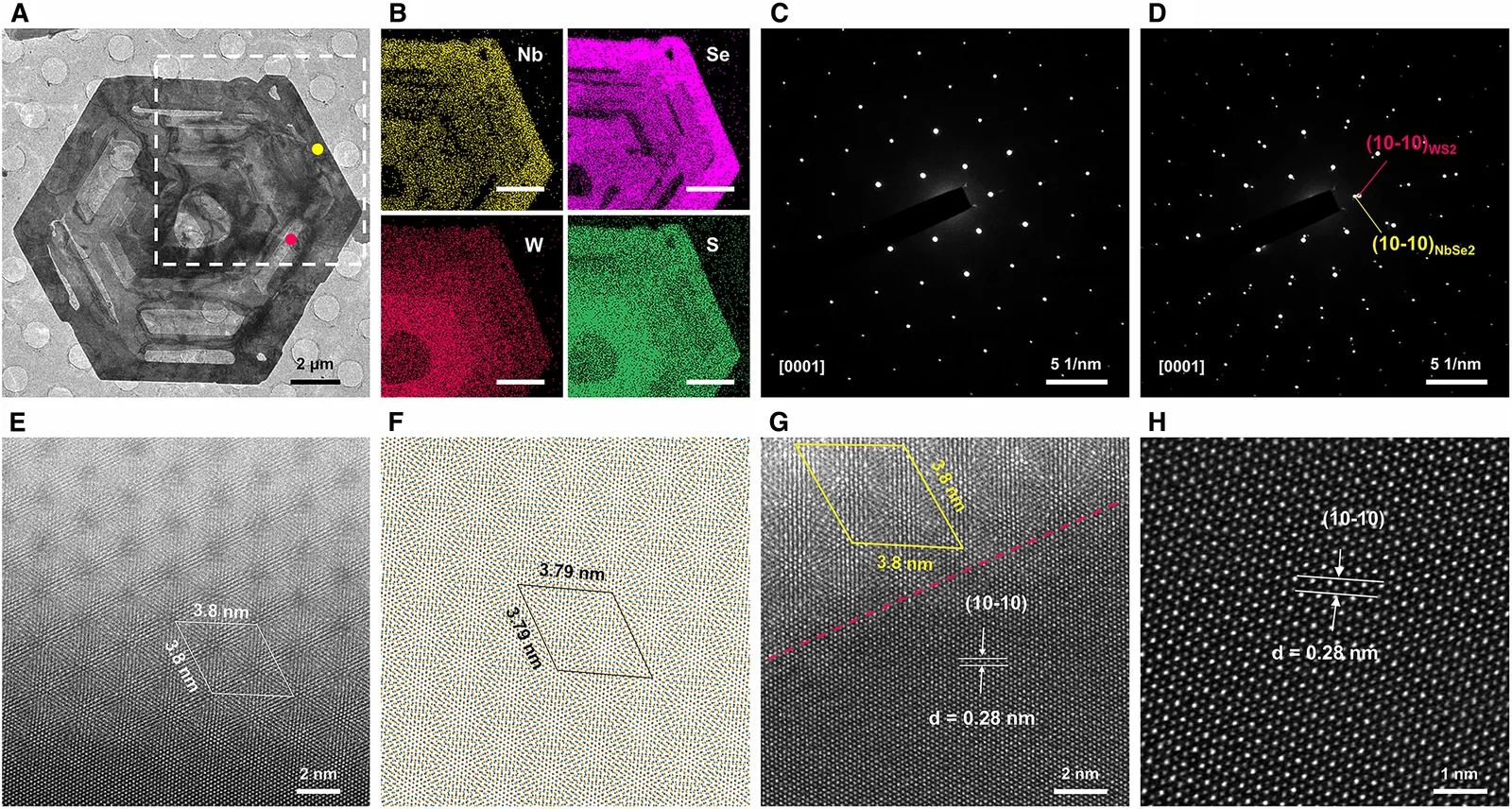
Planar-view STEM characterization of NbSe_2_/Spiral WS_2_ vdWHs. (**A**) Planar-view HAADF-STEM image of a 2D NbSe_2_/Spiral WS_2_ vdWH. The epitaxial region is indicated by a yellow dot, while the underlying spiral growth of WS_2_ is marked with a red dot. (**B**) EDS elemental mapping of the 2D NbSe_2_/Spiral WS_2_ vdWH [white dashed region in (A)], showing the distribution of Nb, Se, W, and S. Scale bars: 2 μm. (**C** and **D**) SAED patterns acquired from the regions labeled by the red and yellow dots in (A), respectively. (**E**) High-magnification HAADF-STEM image of the epitaxial regions. (**F**) Atomic model illustrating the stacking configuration of the 2D NbSe_2_/Spiral WS_2_ vdWH. (**G** and **H**) High-magnification HAADF-STEM images of the heterostructure interface (G) and the spirally grown WS_2_ (H).

### Multiscale cross-sectional characterization of m-TMDs/Spiral s-TMDs vdWHs

Taking advantage of the full-coverage nature of TaSe_2_ epitaxial growth, cross-sectional samples were prepared from the edge of the TaSe_2_/Spiral WS_2_ vdWH (fig. S9) and characterized across multiple scales, from mesoscopic to atomic (Fig. 3, A to D). At the mesoscopic scale (Fig. 3, A and B, and fig. S10), the upper TaSe_2_ layer is thicker and exhibits alternating light and dark vertical stripes distributed throughout the cross-sectional sample. These stripes are likely caused by stress induced by the underlying WS_2_ surface structure (*45*, *46*). At the atomic scale, the WS_2_ beneath the dark streaks of TaSe_2_ displays a continuous cross-layer joint structure, indicative of SDD growth, rather than a simple layer-by-layer (LbL) arrangement (Fig. 3, C and D) (*47*, *48*). Observations of WS_2_ growth on the substrate surface reveal both step-guided growth (Fig. 3, C and D) and curved growth (Fig. 3, E and F). These nanoscale steps and curved surfaces provide the necessary conditions for SDD growth, specifically out-of-plane displacement. The growth shown in Fig. 3F is more complicated, starting with an initial layer curved along the substrate surface (red dashed line), followed by LbL growth with topological scars (blue dashed line), and ultimately resulting in SDD growth (yellow dashed line) (fig. S11). Measurement of the distance between the six atomic layers on the left (4.04 nm) and right (4.14 nm) sides of Fig. 3F exhibits stretching and compression between the layers, consistent with edge dislocation characteristics. Therefore, combined with the alternating mesoscopic stripe patterns, we propose an MSDD growth mechanism for spiral growth, with an estimated dislocation linear density of 10^6^ cm^−1^, derived from stripe spacing measurements. Finally, atomic-level characterization of epitaxially grown TaSe_2_ (Fig. 3G) shows LbL growth without cross-layer joints. These findings suggest that the steps or curved surfaces of the substrate trigger the formation of screw dislocations, potentially involving edge dislocations. While epitaxial TaSe_2_ grows via LbL, spirally grown WS_2_ follows the MSDD growth mechanism (Fig. 3H).

**Fig. 3.**
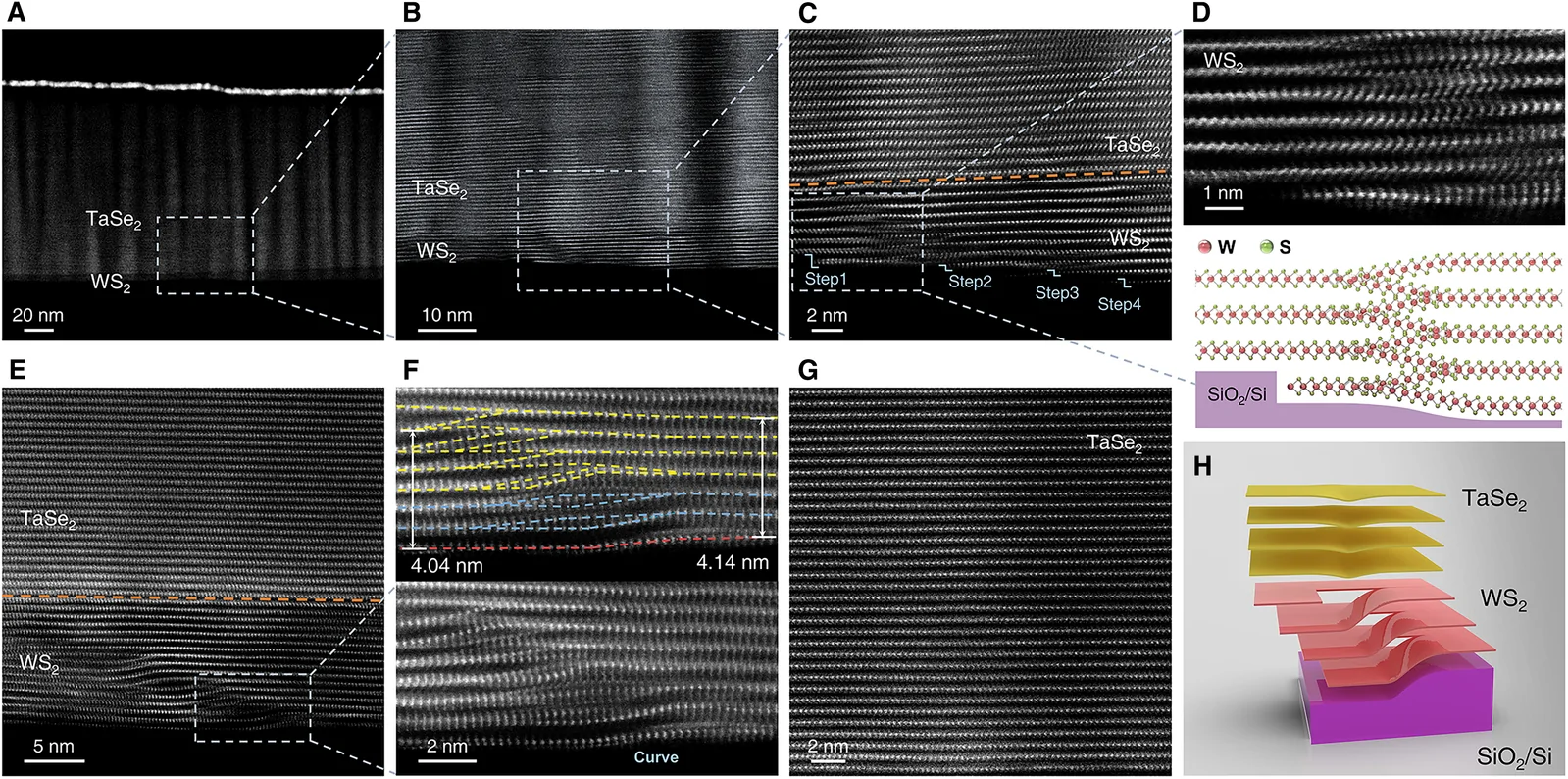
Multiscale cross-sectional analysis of TaSe_2_/Spiral WS_2_ vdWHs. (**A** to **D**) Cross-sectional HAADF-STEM images of a TaSe_2_/Spiral WS_2_ vdWH, ranging from mesoscopic to atomic scale. The orange dashed line in (C) indicates the location of the heterostructure interface. The diagram in (D) illustrates the atomic model of SDD growth of WS_2_. (**E** and **F**) Cross-sectional HAADF-STEM images of the same TaSe_2_/Spiral WS_2_ on a nanoscale curved substrate. The orange dashed line in (E) marks the position of the heterostructure interface. (**G**) High-resolution HAADF-STEM image of the upper TaSe_2_ layer. (**H**) Schematic illustration of the stacking mechanism, highlighting SDD growth of WS_2_ and LbL growth of TaSe_2_. Created with Blender.

### Mechanisms of MSDD growth

2D materials exhibit extreme aspect ratios of up to 10^9^ or more, necessitating growth rates that differ by factors of millions or even billions between the in-plane (parallel to the 2D basal plane) and out-of-plane (normal to the 2D basal plane) directions (*49*, *50*). This pronounced growth anisotropy is crucial for understanding their morphological evolution. The substrates used for 2D spiral growth in this study were typically SiO_2_ (*11*–*14*, *51*–*54*), which are rarely atomically flat. Their surfaces commonly exhibit nanoscale undulations, including features such as bumps, pleats, grooves, and steps (Figs. 3 and 4A) (*17*–*20*). AFM measurements confirmed sub-nanometer surface roughness (fig. S12). As a control, growth under the same conditions on atomically flat sapphire substrates yielded conventional 2D flakes (fig. S13), demonstrating the critical role of substrate topography in modulating the growth mode. 2D spiral growth begins with nucleation on the substrate (Fig. 4B). Benefiting from the extremely high growth anisotropy, the initially nucleated crystal rapidly undergoes conformal in-plane expansion while strictly maintaining its crystallographic orientation (Fig. 4C). As growth progresses over curved surfaces, the lattice bends, requiring the overcoming of energy barriers that reduce the growth rate. Growth preferentially occurs on surfaces with low Gaussian curvature, whereas high-curvature regions frequently develop defects or cracks (Fig. 4F) (*55*–*59*). Continued growth along curved surfaces induces out-of-plane displacements, enabling new layers to stack laterally on top of the initial layer (Fig. 4E). Thermodynamically stable stacking configurations, such as 2H stacking (with a 60° interlayer rotation) and 3R stacking (requiring interlayer slip), dominate in TMDs due to their small energy differences (*60*). First-principles calculations of gliding energy (fig. S14A) suggest that stacked growth initiates with random stacking, transitioning to 3R stacking as layers expand, driven by the competition between slip and curvature-induced strain energy (fig. S15). This self-perpetuating growth mechanism, transitioning from the initial layer to the new layer, is identified as the SDD growth mechanism (Fig. 4D). SDD growth can be classified based on the involvement of edge dislocations, which lead to the formation of mixed dislocations, as well as the magnitude of out-of-plane displacements (fig. S16). Adjacent SDD growth fronts subsequently merge, establishing the foundation for macroscopic spiral growth, thereby defining the MSDD growth mechanism (Fig. 4G). Through phase-field simulations, we have fully reproduced the entire process, from initial nucleation and conformal in-plane expansion to the final curvature-induced growth of multiple screw dislocations, thereby providing quantitative support for the proposed MSDD growth mechanism (fig. S17).

**Fig. 4.**
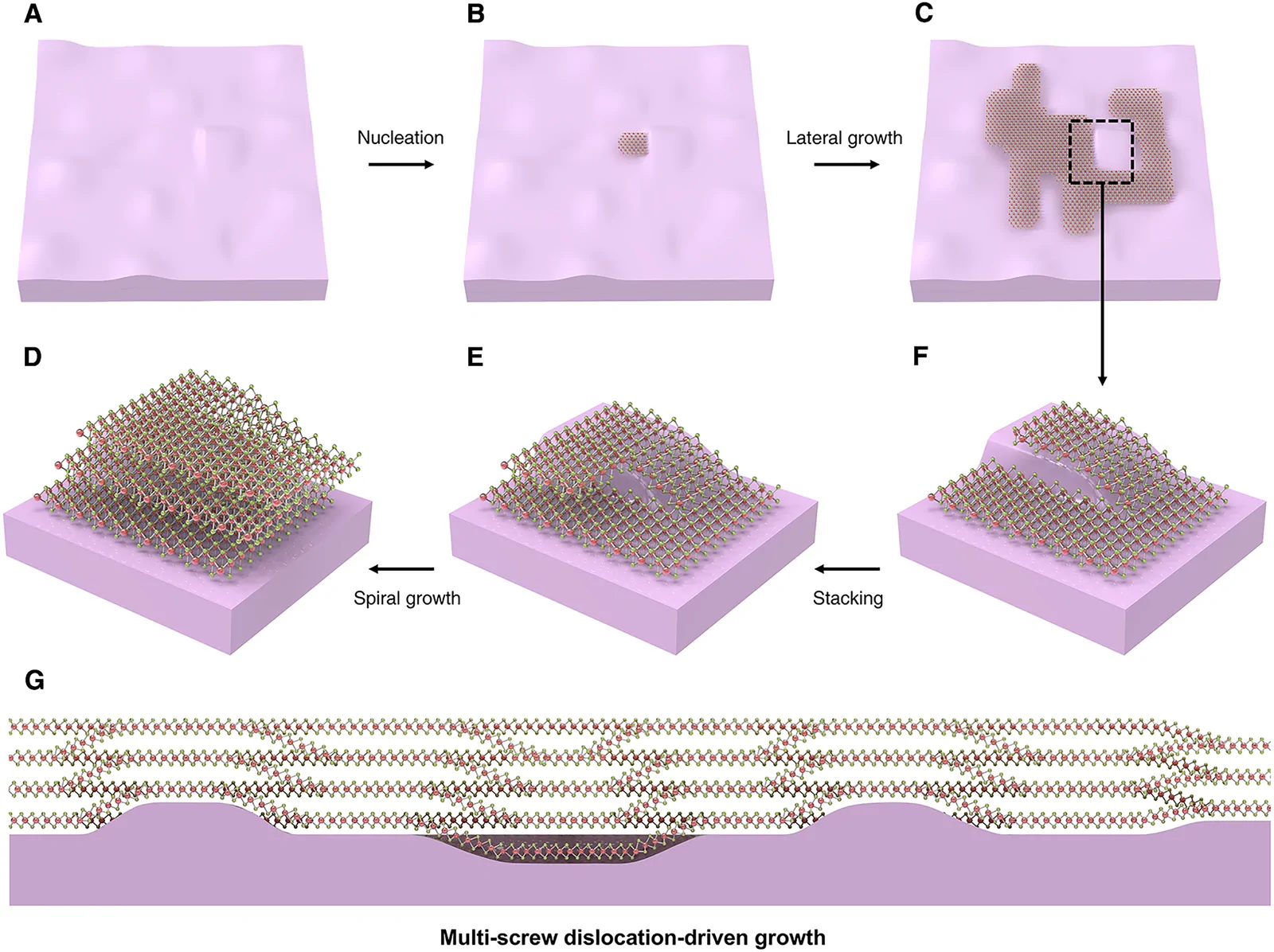
Schematic representation of MSDD growth. (**A**) Substrate for 2D material growth, characterized by nanoscale surface undulations. (**B** and **C**) Schematic illustrations of nucleation (B) and growth (C) of a 2D material on the substrate. (**D** and **E**) Out-of-plane displacement facilitates SDD growth (D) and lateral stacking (E) on the initial layer. (**F**) Schematic diagram illustrating a 2D material growing along a curved surface, forming a crack, and resulting in out-of-plane displacement. (**G**) Schematic illustration of MSDD growth. Created with Blender.

Combined with multiscale STEM studies, it is confirmed that WS_2_ follows an MSDD growth mechanism, whereas TaSe_2_ adopts an LbL growth mechanism. To further elucidate these two mechanisms, we evaluated the bonding strengths of WS_2_ and TaSe_2_ separately through first-principles calculations. The results reveal that the bonding strength of Ta-Se (∼3.082 eV per bond) is comparable to that of W-S (∼3.073 eV per bond) (fig. S14, B and C), indicating similar bond-breaking capabilities. Therefore, it is reasonable to conclude that in MSDD-grown WS_2_, the out-of-plane dislocations offset the substrate undulations, resulting in a flatter WS_2_ surface for the epitaxial growth of TaSe_2_. The dislocations at the surface of WS_2_ (dislocation ends at the surface) are insufficient to cause Ta-Se bond breaking but instead generate mesoscopic stress stripes.

### Interlayer stacking reconstruction

The stacking structure of m-TMDs/Spiral s-TMDs vdWHs was investigated using a representative cross-sectional HAADF-STEM image (Fig. 5A). Enlarged views of TaSe_2_ (Fig. 5B) and WS_2_ (Fig. 5C) from Fig. 5A confirm that both stacks belong to the 3R phase. In this phase, the unit cell consists of three distinct layers, all aligned in the same direction, with adjacent layers sliding by $a/\sqrt{3}$ along the in-plane armchair direction, where a represents the lattice constant (fig. S18). Figure 5D highlights the interface of the TaSe_2_/Spiral WS_2_ vdWH, revealing a sharp atomic boundary without substantial atomic mixing. However, anomalous stacking phenomena are observed in WS_2_ (Fig. 5E), including interlayer slipping and variations in interlayer distance. Planar-view HAADF-STEM images of WS_2_ further reveal two distinct anomalous stacking phases, labeled 2RA (Fig. 5, F and G) and 2RB (Fig. 5, H and I). The unit cell of the 2R phase consists of two distinct layers, both aligned in the same direction. The sliding distances of adjacent layers along the in-plane armchair direction are measured as 0.28 nm and 0.13 nm for the 2RA and 2RB phases, respectively, compared to approximately 0.18 nm for the 3R stacking. The 2R phase is not thermodynamically stable but can be explained by the interaction of multiple screw dislocations during growth. According to the MSDD growth mechanism, all new layers formed via screw dislocations originate from the same initial layer and maintain identical crystal orientations. The merging of adjacent layers does not result in crystal boundaries but induces localized lattice stresses due to differences in out-of-plane displacements before merging. Additionally, screw dislocations exert a pinning effect, driving interlayer reconstruction, manifesting as slipping and variations in interlayer distance within the new layers between screw dislocations (fig. S19). When the difference in out-of-plane displacements between adjacent layers exceeds a critical threshold, it results in the formation of a topological scar structure (fig. S20). Due to the fundamental limitations of cross-sectional TEM characterization (note S1), the precise characterization of complex screw dislocations has always been challenging. The contrast of screw dislocations is highly dependent on imaging conditions, such as focus, sample orientation, and astigmatism correction (*22*). In particular, under certain imaging conditions, screw dislocation regions may exhibit the artifact of layered growth (fig. S21). Furthermore, the interaction of multiple screw dislocations further exacerbates this issue (note S2). Consequently, it is nearly impossible to image all screw dislocations with optimal contrast in a single STEM image; in practice, analysis is typically confined to specific regions of interest. Based on this, our analysis of multi-screw dislocation systems is strictly grounded in observable, reproducible, and interpretable phenomena.

**Fig. 5.**
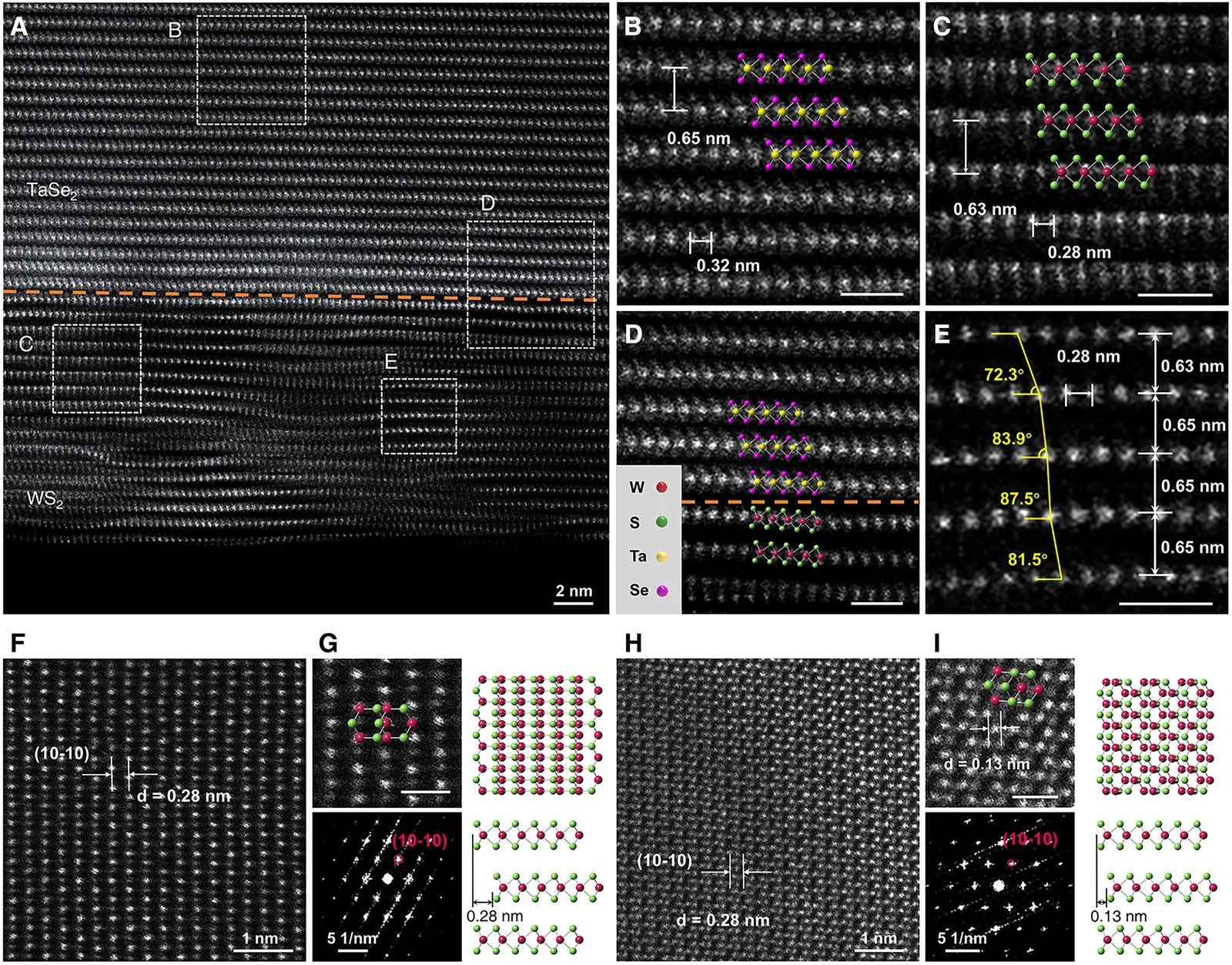
Atomic-scale interlayer stacking reconstruction in TaSe_2_/Spiral WS_2_ vdWHs. (**A**) Cross-sectional HAADF-STEM image of a TaSe_2_/Spiral WS_2_ vdWH, with the orange dashed line indicating the location of the heterostructure interface. (**B** to **E**) Magnified views of selected regions from (A), revealing atomic-resolution details of the interlayer stacking configuration. Scale bars: 1 nm. (**F**) HAADF-STEM image of 2RA-WS_2_. (**G**) High-magnification HAADF-STEM image of 2RA-WS_2_, accompanied by the corresponding fast Fourier transform (FFT) pattern and atomic schematic. Scale bars: 0.5 nm. (**H**) HAADF-STEM image of 2RB-WS_2_. (**I**) High-magnification HAADF-STEM image of 2RB-WS_2_, accompanied by the FFT pattern and atomic schematic. Scale bars: 0.5 nm.

### Mechanistic insights into spiral morphologies

To elucidate the macroscopic morphological consequences of MSDD growth, we analyzed a cross-sectional sample from the central region of a TaSe_2_/Spiral WS_2_ vdWH (Fig. 6A and fig. S22). Low-magnification HAADF-STEM imaging shows a protruding feature (∼500 nm in diameter) exhibiting non-Euclidean surface morphology (Fig. 6B). Higher-magnification analysis of the protrusion apex displays alternating bright-dark contrast in the WS_2_ layer, accompanied by an anomalous lateral linear defect (Fig. 6C). Atomic-scale characterization (white dashed box, Fig. 6C) identifies a 7° $\left[1\bar{2}10\right]$ low-angle grain boundary (LAGB) consisting of aligned dislocations with a Burgers vector of 1/3[0001] (Fig. 6D). Crystallographic analysis of WS_2_ in Fig. 6D uncovers three competing growth domains (G1, G2, and G3) with misorientations that induce interlayer stacking reconstructions and localized strain accumulation. During the out-of-plane growth of MSDD, while the intermediate G2 domain initially accommodates orientation transitions, its progressive width reduction intensifies lattice strain and curvature gradients, suppressing growth in both G2 and G3 domains (*61*). Consequently, the dominant lateral expansion of the G1 domain across the G3 surface leads to LAGB formation (fig. S23). Notably, the WS_2_ grain indicated by the arrow in Fig. 6A shares the same crystallographic orientation as the G1 domain, and correlative multi-scale STEM analysis confirmed its origin from the G1 domain. Atomic-resolution HAADF-STEM imaging (Fig. 6, E and F) resolves screw dislocations in both G1 and G3 domains. However, the misalignment between the incident electron beam and the crystalline zone axis limits the precise measurement of screw dislocations. These constraints currently preclude complete simultaneous characterization of multiple screw dislocations. Our findings indicate that the kinetic competition among multiple screw dislocations can considerably influence the macroscopic morphology.

**Fig. 6.**
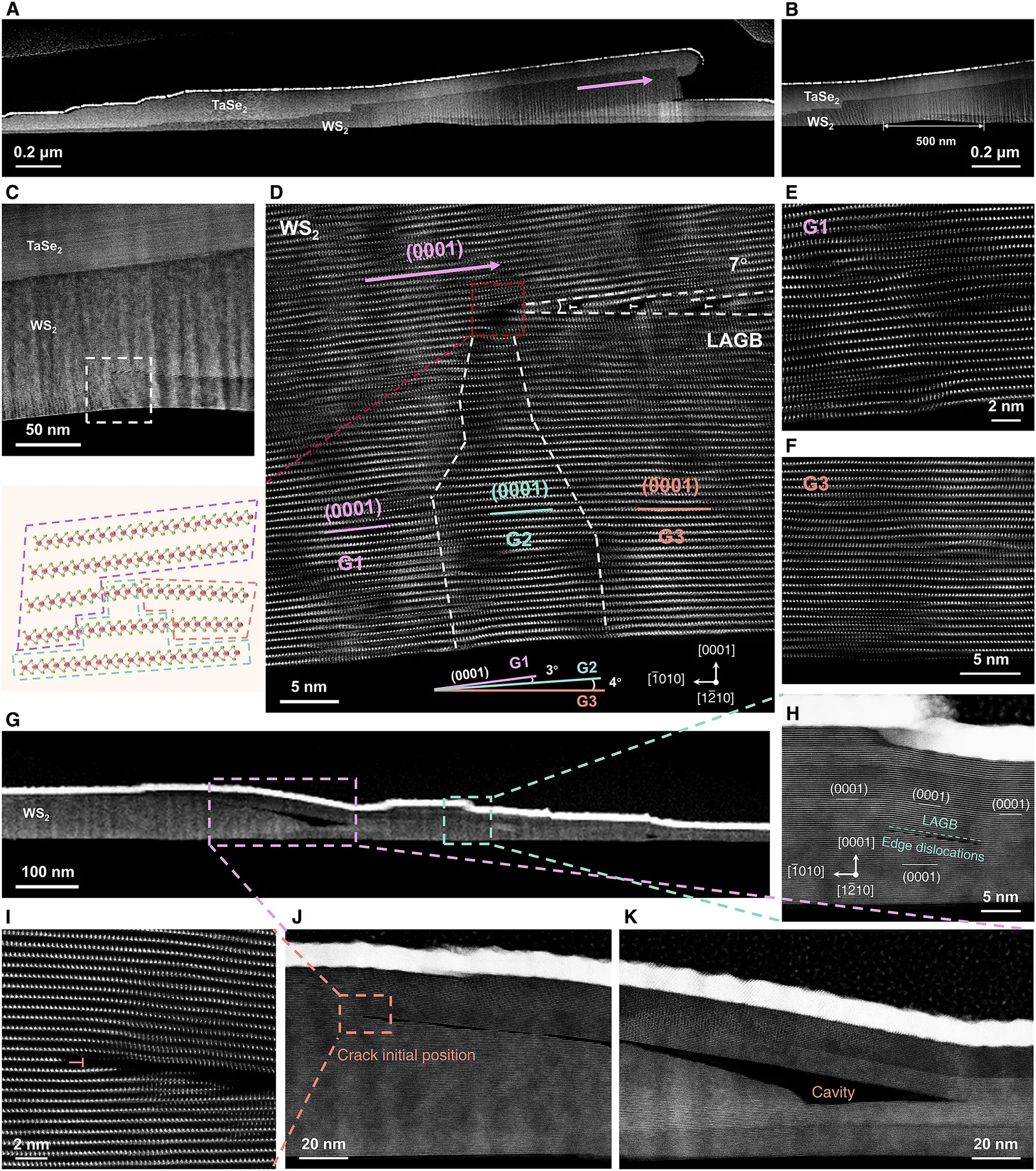
Multiscale observations of competitive growth mechanism. (**A** to **C**) Cross-sectional HAADF-STEM images of the central region of the TaSe_2_/Spiral WS_2_ vdWH at different magnifications. (**D**) A magnified view of the STEM image corresponding to the white dashed area in (C). The inset in the lower left corner provides an enlarged schematic representation of the region highlighted by the red dashed box in (D). (**E** and **F**) Further magnified views of the G1 and G3 grains, respectively. (**G**) Cross-sectional HAADF-STEM image of the central region of spirally grown WS_2_. (**H**) A detailed view of the step region indicated in (G). (**I** to **K**) Successively enlarged views of the crack and cavity area within (G).

Cross-sectional analysis of the central region of a pure WS_2_ sample (Fig. 6G and fig. S24) revealed similar growth features: while the substrate surface shows no distinct non-Euclidean protrusions, the WS_2_ layer clearly demonstrates a competitive growth mechanism of multiple screw dislocations, as evidenced by well-defined step features (mint green dashed lines) and internal cavities (light magenta dashed lines). High-resolution examination of the step region (Fig. 6H) identifies a LAGB composed of aligned dislocations. Crystallographic analysis reveals that grains below the LAGB maintain a uniform orientation, whereas those above the LAGB show notable tilting. The cavity evolution sequence (Fig. 6, I to K) documents the complete dynamic process from dislocation formation, through crack propagation, to final cavity development. These findings provide direct evidence that the spiral morphology in 2D materials originates from the competitive growth mechanism of multiple screw dislocations.

The competitive growth mechanism of multiple screw dislocations is further validated by Raman spectroscopy. The Raman mapping of the defect-related *LA*(**M**) mode in spirally grown WS_2_ demonstrates that the dislocation density at the sample edges is pronouncedly higher than that in the central region, indicating a continuous decrease in dislocation density as growth proceeds (Fig. 1L and fig. S2F) (*39*, *40*). A plausible explanation for this phenomenon is the dislocation coverage mechanism arising from the competitive growth of multiple screw dislocations. In the thinner edge regions, WS_2_ grows primarily via the MSDD mechanism on the substrate, generating multiple screw dislocations; whereas intense growth competition occurs among multiple screw domains in the central region. During this process, screw domains with growth advantages expand laterally, covering adjacent screw domains with slower growth rates, thereby cutting off their material supply and leading to growth termination (fig. S25). Due to the lower dislocation density of the covering layer, the signals from the underlying dislocations are substantially attenuated, manifesting in Raman mapping as an apparent continuous reduction in dislocation density. This mechanism is supported by mesoscale experimental evidence, including the grain boundaries, cracks, and cavities observed in our samples.

Based on these findings, we propose a multi-scale evolutionary model for spiral growth in 2D materials, driven by MSDD mechanism (Fig. 7). Initially, nanoscale surface undulations of the substrate trigger the MSDD growth mode. Subsequently, adjacent screw dislocations achieve coordinated growth through interlayer stacking reconstruction. This is followed by the kinetic competition among multiple screw dislocations, ultimately leading to the formation of macroscopic spiral morphologies. The MSDD mechanism represents an important extension and refinement of the classical BCF theory and the traditional SDD model. The BCF theory elucidates the role of screw dislocations as persistent growth steps under low supersaturation conditions, while the SDD model extends this principle to the anisotropic growth of nanomaterials. The key breakthrough of this study lies in the approach of constructing m-TMDs/Spiral s-TMDs vdWHs and employing cross-sectional characterization, which enables robust multi-scale observation of screw dislocations spanning from atomic to macroscopic scales. This advancement shifts the research perspective from individual dislocation behavior to the cooperative origin and collective dynamics of multiple dislocations. Consequently, the MSDD mechanism establishes a systematic theoretical framework that connects atomic-scale defect origins with the regulation of macroscopic morphology, providing a new paradigm for the design and control of spiral growth in 2D materials.

**Fig. 7.**
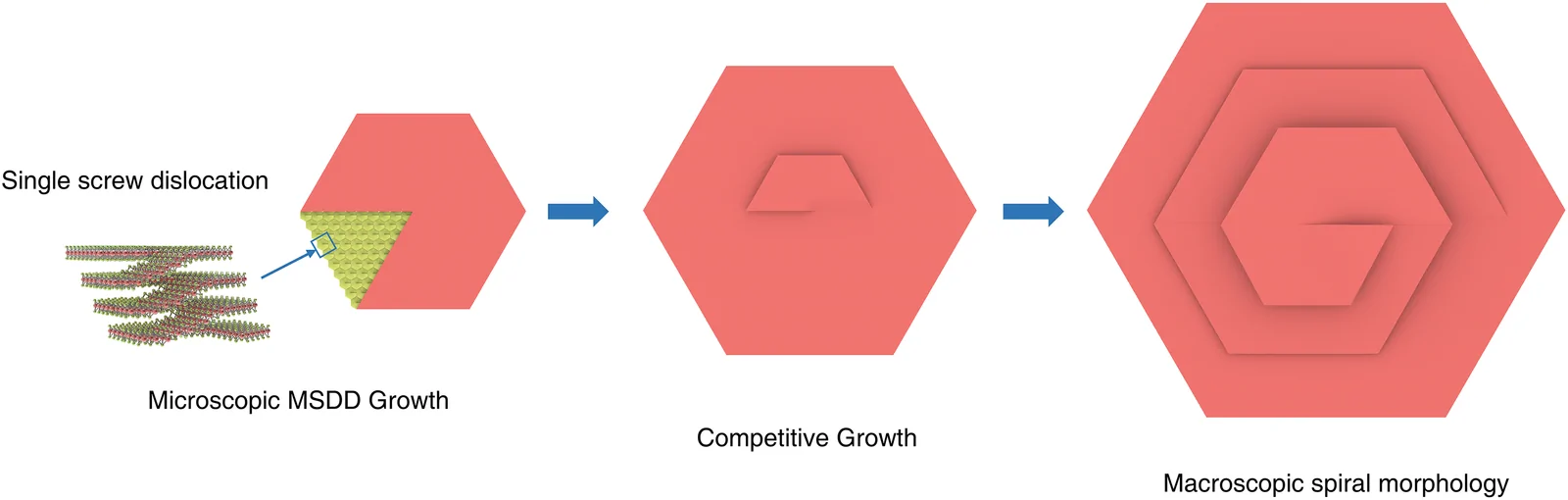
Multiscale schematic diagram of 2D spiral growth. Nanoscale undulations on the substrate initially induce MSDD growth. Subsequently, competitive interactions between MSDD domains promote lateral expansion, ultimately resulting in macroscopic spiral morphologies. Created with Blender.

## DISCUSSION

In summary, we present a multiscale study of SDD growth mechanisms through the synthesis of m-TMDs/s-TMDs spiral vdWHs, including TaSe_2_/Spiral WS_2_, TaSe_2_/Spiral WSe_2_, TaSe_2_/Spiral MoS_2_, NbSe_2_/Spiral WS_2_, and NbSe_2_/Spiral MoS_2_. Notably, the epitaxial m-TMDs overlayers preserve Spiral s-TMDs microstructures during FIB sample preparation, enabling robust atomic-resolution imaging of screw dislocations. Furthermore, stress stripes observed in cross-sectional samples of the heterostructures elucidate the MSDD growth mechanism underlying spiral growth. Multiscale STEM analysis provides comprehensive insights into the spiral growth process, including screw dislocation formation, interlayer stacking reconstruction, competing growth mechanisms, and the resulting macroscopic morphology. This framework extends beyond TMDs to other dislocation-mediated growth systems, offering a general paradigm for designing advanced stacked materials with programmable interlayer geometries. By harnessing screw dislocations as nanoscale “growth engines.” we unlock new avenues for fabricating materials with engineered quantum phases and strain-tuneable functionalities.

## MATERIALS AND METHODS

### Sample preparation

The spiral growth of 2D TMDs and m-TMDs/Spiral s-TMDs vdWHs was carried out in a 1-inch-diameter quartz tube, housed within a furnace approximately 36 cm in length. Argon (Ar) or an argon-hydrogen (Ar/H_2_) mixture was used as the carrier gas. A homemade quartz boat containing precursor powders was positioned at the center of the tube. The substrate was placed face-down at a 45° tilt angle above the precursor powders, using either a SiO_2_ (300 nm)/Si substrate treated with piranha solution or a sapphire substrate. All substrates were purchased from the Tianjin Semiconductor Technology Research Institute. A ceramic boat with sulfur (S) or selenium (Se) powder was placed upstream of the furnace, maintained at 300°C and 450°C, respectively, with distances of approximately 18 cm and 16 cm from the precursor boat. To purge the system of oxygen, 400 sccm of Ar gas was flowed for 20 minutes. All reactions were conducted at atmospheric pressure with a heating rate of 25°C/min, followed by natural cooling to room temperature. All materials were purchased from Alfa Aesar, with a purity of >99%.

Spiral growth of WS_2_: A mixture powder of 0.5 mg NaCl and 50 mg WO_3_ was placed at the center of the quartz tube. The furnace was heated to 1000°C within 50 minutes, and the growth proceeded for 15 minutes under a 40 sccm flow of Ar.

Spiral growth of WSe_2_: The spiral growth of WSe_2_ followed a procedure similar to that of WS_2_. The growth temperature was set to 950°C, with an Ar/H_2_ (35/5 sccm) flow used as the carrier gas.

Spiral growth of MoS_2_: A mixture of 0.1 mg NaCl and 5 mg MoO_3_ was used. The furnace was heated to 750°C within 30 minutes, and growth lasted for 10 minutes under a 40 sccm flow of Ar.

Growth of TaSe_2_/Spiral WS_2_ vdWHs: The substrate with spirally grown WS_2_ was transferred to another quartz tube. A ceramic boat containing Ta_2_O_5_ (∼40 mg), Ta (∼20 mg) and NaCl (∼5 mg) powder was positioned at the center of the furnace. Se (∼300 mg) powder was placed upstream (∼25 cm from the heating zone center). The WS_2_ surface of the substrate was positioned face down on the precursor powder. Growth was conducted at 750°C for 5 minutes, with Ar/H_2_ (35/5 sccm) as the carrier gas.

Growth of TaSe_2_/Spiral WSe_2_ vdWHs: This growth followed the same method as that of TaSe_2_/WS_2_, using a WSe_2_ spiral substrate. A mixture of Ta_2_O_5_ (∼40 mg), Ta (∼20 mg), and NaCl (∼5 mg) served as the precursor. Growth was performed at 750°C for 10 minutes, with Ar/H_2_ (55/5 sccm) as the carrier gas.

Growth of TaSe_2_/Spiral MoS_2_ vdWHs: The substrate with spirally grown MoS_2_ was used as the epitaxial substrate. A mixture of Ta_2_O_5_ (∼40 mg), Ta (∼20 mg), and KCl (∼5 mg) was used as the precursor for the epitaxial growth. Growth was performed at 700°C for 10 minutes, with Ar/H_2_ (55/5 sccm) as the carrier gas.

Growth of NbSe_2_/Spiral WS_2_ vdWHs: The substrate with spirally grown WS_2_ was used as the epitaxial substrate. A mixture of Nb_2_O_5_ (∼40 mg), Nb (∼20 mg), and KCl (∼5 mg) was used as the precursor for the epitaxial growth. Growth was carried out at 750°C for 15 minutes, with Ar/H_2_ (55/5 sccm) as the carrier gas.

Growth of NbSe_2_/Spiral MoS_2_ vdWHs: The substrate with spirally grown MoS_2_ served as the epitaxial substrate. A precursor mixture of Nb_2_O_5_ (∼40 mg), Nb (∼20 mg), and KCl (∼5 mg) was used for the epitaxial growth. Growth was performed at 700°C for 10 minutes, with Ar/H_2_ (45/5 sccm) as the carrier gas.

### Transfer of samples

To prepare the as-grown m-TMDs/Spiral s-TMDs vdWHs for analysis, a thin film of poly (methyl methacrylate) (PMMA, 4 wt %, 950 K molecular weight) solution was spin-coated onto the samples at 1000 rpm for 1 minute. The samples were then heated on a hot plate at 110°C for 10 minutes. Strips (∼1 mm wide) of PMMA along the substrate edges were carefully removed using a blade, and the substrate was subsequently immersed upside down in deionized water for 2 hours to facilitate the extraction of the PMMA/heterostructure films. The detached films were transferred onto a holey carbon film supported on copper grids. The samples were then dried in an oven at 60°C for 2 hours to remove residual water and enhance adhesion between the PMMA/heterostructure film and the copper grid. Finally, the PMMA layer was dissolved in dichloromethane for 30 minutes.

### Materials characterization

Optical images were captured using a Nikon inverted microscope Eclipse Ti-U with a CCD of Nikon Digital Sight. SEM images were acquired with a Hitachi S-4800 field-emission microscope operating at 15 kV. Raman spectra and mappings were performed with the Renishaw InVia Raman microscope and Nanophoton Raman 11 Microscope. AFM characterizations were obtained using a Bruker Dimension FastScan. STEM images and EDS maps were acquired using an aberration-corrected JEM-ARM300F operating at 300 kV. Cross-sectional samples for STEM were fabricated using an FEI Helios-600i FIB.

### Theoretical calculation

Density functional theory (DFT) calculations were performed to study the gliding energy and bonding strength of the TMDs, in which the Vienna Ab initio Simulation Package (VASP.6.3.2) (*62*, *63*) was used with a projector augmented wave basis (*64*) and Perdew-Burke-Ernzerhof parameterized generalized gradient approximation exchange-correlation functional (*65*). For all the calculations, the kinetic energy cutoff for electronic plane wave expansion was set to 400 eV. Γ-centred 15 × 15 × 1 and 12 × 12 × 1 k-points grids were used to sample the first Brillouin zone of the WS_2_ and TaSe_2_ unit cell and supercell, respectively. The crystal orbital Hamilton populations (COHP) was calculated using the LOBSTER package (*66*) to estimate the bond strength. The integration of partial COHP for the bonding state (below the Fermi level) was conducted to estimate the strength of W-S and Ta-Se bonding due to their identical bonding configuration.

### Phase-field simulation method

To quantitatively investigate the morphological evolution of TMDs during vapor-phase deposition, we adopt a phase-field framework that couples interfacial dynamics with surface diffusion of adatoms (*67*, *68*). The model is formulated in dimensionless units and consists of two coupled fields: a structural order parameter ϕ(x,y,t) that distinguishes solid from vapor phases, and a supersaturation field ξ(x,y,t) representing the local concentration of adsorbed precursors (*69*, *70*). The evolution of the order parameter is governed by Eq. 1

$$\tau_{\phi }\left(\theta \right)\frac{\partial \phi }{\partial t}=W^{2}\nabla^{2}\phi +\text{sin}\left[\pi \left(\phi -\phi_{s}\right)\right]+\lambda \xi \left(1+\text{cos}\left[\pi \left(\phi -\phi_{s}\right)\right]\right)$$ (1)

where *W* sets the interface width, λ controls the strength of the growth driving force, and $\tau_{\phi }\left(\theta \right)$ is an orientation-dependent kinetic coefficient. The reference field $\phi_{s}\left(x,y\right)$ encodes the topological constraint imposed by screw dislocations (*71*, *72*). For a system containing multiple dislocations at positions (*x_i_, y_i_*) with Burgers vectors **b***_i_* = *s_i_*h (*s_i_* = ±1). $\phi_{s}$ is constructed as

$$\phi_{s}\left(x,y\right)=\sum_{i}\left[s_{i}h\left(\frac{\text{atan}2\left(y-y_{i},x-x_{i}\right)+\varphi_{i}}{\pi }+1\right)\text{mod}2-1\right]$$ (2)

To account for the six-fold rotational symmetry of TMDs, the mobility $\mu =1/\tau_{\phi }\left(\theta \right)$μ is taken as anisotropic

$$\mu \left(\theta \right)=\left(\mu_{0}+\mu_{1}\right)|\text{sin}\left(3\theta \right)|+\left(\mu_{0}+\mu_{2}\right)|\text{cos}\left(3\theta \right)|-\mu_{0}$$ (3)

where $\theta =\text{atan}2\left(\partial_{y}\phi ,\partial_{x}\phi \right)$ is the local interface normal angle. This form reproduces the expected truncated hexagonal equilibrium shape. The supersaturation field evolves according to a reaction-diffusion equation

$$\frac{\partial \xi }{\partial t}=D\nabla^{2}\xi -\frac{\xi }{\tau_{s}}+F\left(\phi \right)-\frac{1}{2}\frac{\partial \phi }{\partial t}$$ (4)

Here, *D* is the surface diffusivity, $\tau_{s}$ is the characteristic desorption time, and the source term F(ϕ) models the deposition flux. Critically, we distinguish between growth on bare substrate and on existing film by setting a piecewise deposition flux with F_sub_ < F_film_ (Eq. 5), reflecting the experimentally observed slower nucleation kinetics of the first monolayer due to stronger substrate interactions.

$$F\left(\phi \right)=\left\{ \begin{array}{c} F_{\text{sub}},\;\phi -\phi_{s}<0\\ F_{\text{film}},\;\phi -\phi_{s}>0 \end{array}\right.$$ (5)

Numerically, the equations are solved on a *L* × *L* periodic domain with grid spacing Δx=Δy=0.5. The Laplacian is discretized with second-order central differences, and time integration uses an explicit Euler scheme for Eq. 1 and a semi-implicit Fourier-spectral method for Eq. 4. Specifically, the supersaturation equation is advanced in Fourier space

$$\hat{\xi^{n+1}}=\frac{\hat{\xi^{n}+\Delta t\left(F-\frac{\partial \phi }{\partial t}\right)}}{1+\Delta t\left(Dk^{2}+\tau_{s}^{-1}\right)}$$ (6)

where ^ denotes the discrete Fourier transform and $k^{2}=k_{x}^{2}+k_{y}^{2}$. To maintain numerical stability, the phase field is periodically renormalized. All simulations use the following dimensionless parameters: L=300, Δt=0.01, W=1.5, λ=50, D=50, $\tau_{s}=1$, $F_{\text{film}}=0.12$, $F_{\text{sub}}=0.03$, and anisotropy coefficients $\mu_{0}=2.5$, $\mu_{1}=0.75$, $\mu_{2}=0.25$. These values ensure a sharp interface, pronounced growth anisotropy, and a clear distinction between first-layer and multilayer growth regimes. To simplify the calculations, we used four screw dislocations with identical chirality to simulate a multi-screw-dislocation system.

## Supplementary Materials

### This PDF file includes:

Figs. S1 to S25 Notes S1 and S2 References
