# Supplementary material for: Multiscale insights into screw dislocation-driven growth mechanisms
Source: Sci Adv. 2026 Oct 9;12(41):eaei7996. doi: 10.1126/sciadv.aei7996 (PMC13651847; doi:10.1126/sciadv.aei7996)
Supplement: Supplementary file 1 [file sciadv.aei7996_sm.pdf]

Science Advances
AAAS

Supplementary Materials for

Multiscale insights into screw dislocation-driven growth mechanisms

Peiyu Qiao *et al.*

Corresponding author: Peiyu Qiao, peiyu.qiao@xjtlu.edu.cn; Chun Zhao, chun.zhao@xjtlu.edu.cn;
Xiangmin Meng, mengxiangmin@mail.ipc.ac.cn

Sci. Adv. **12**, eaei7996 (2026)
DOI: 10.1126/sciadv.aei7996

This PDF file includes:

Figs. S1 to S25
Note S1 and S2
References

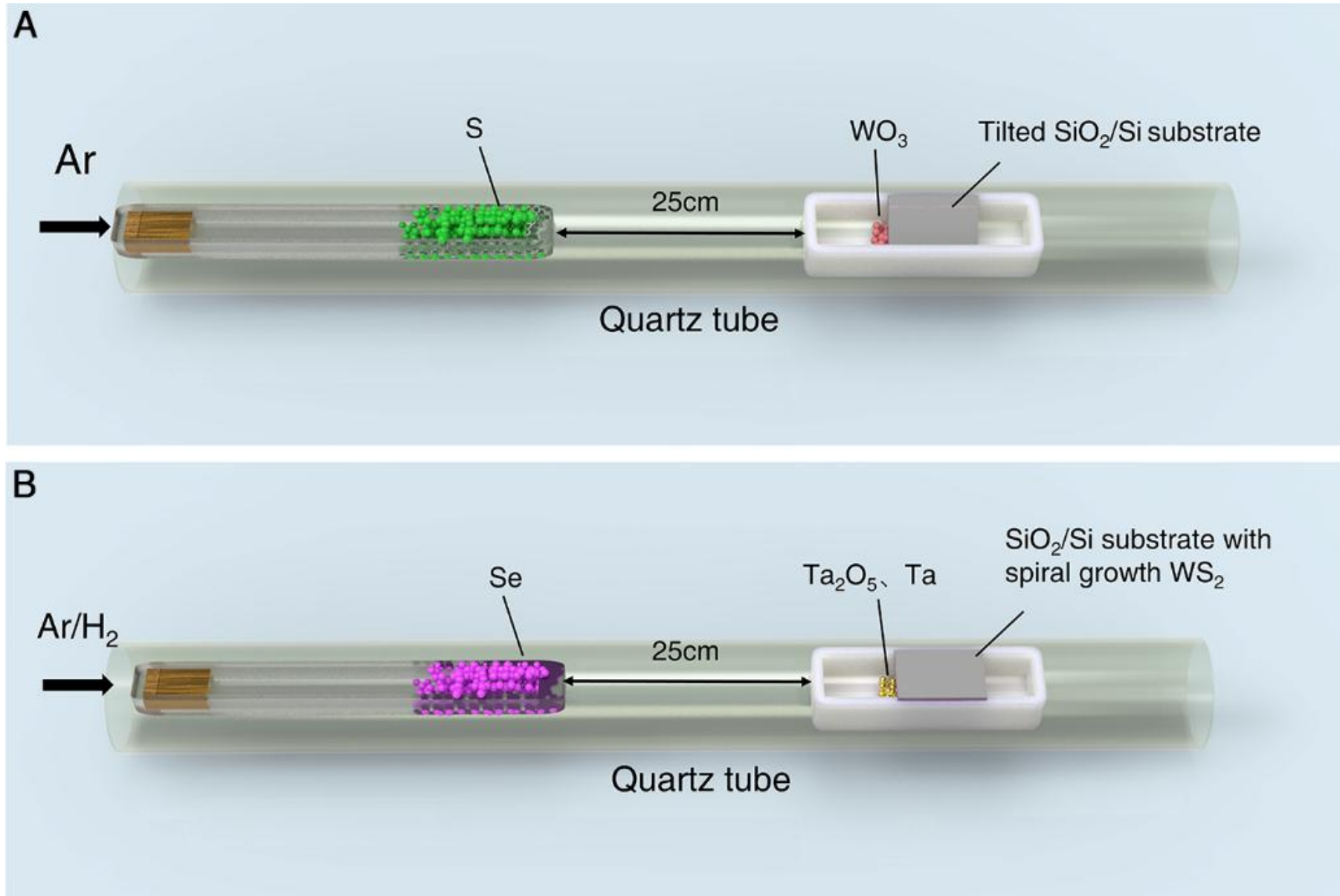

Fig. S1. CVD synthesis schematics. (**A** and **B**) Schematic representation of 2D WS_2 (A) and 2D $TaSe_2$/Spiral WS_2 vdWHs (B) prepared by CVD process. Created with Blender.

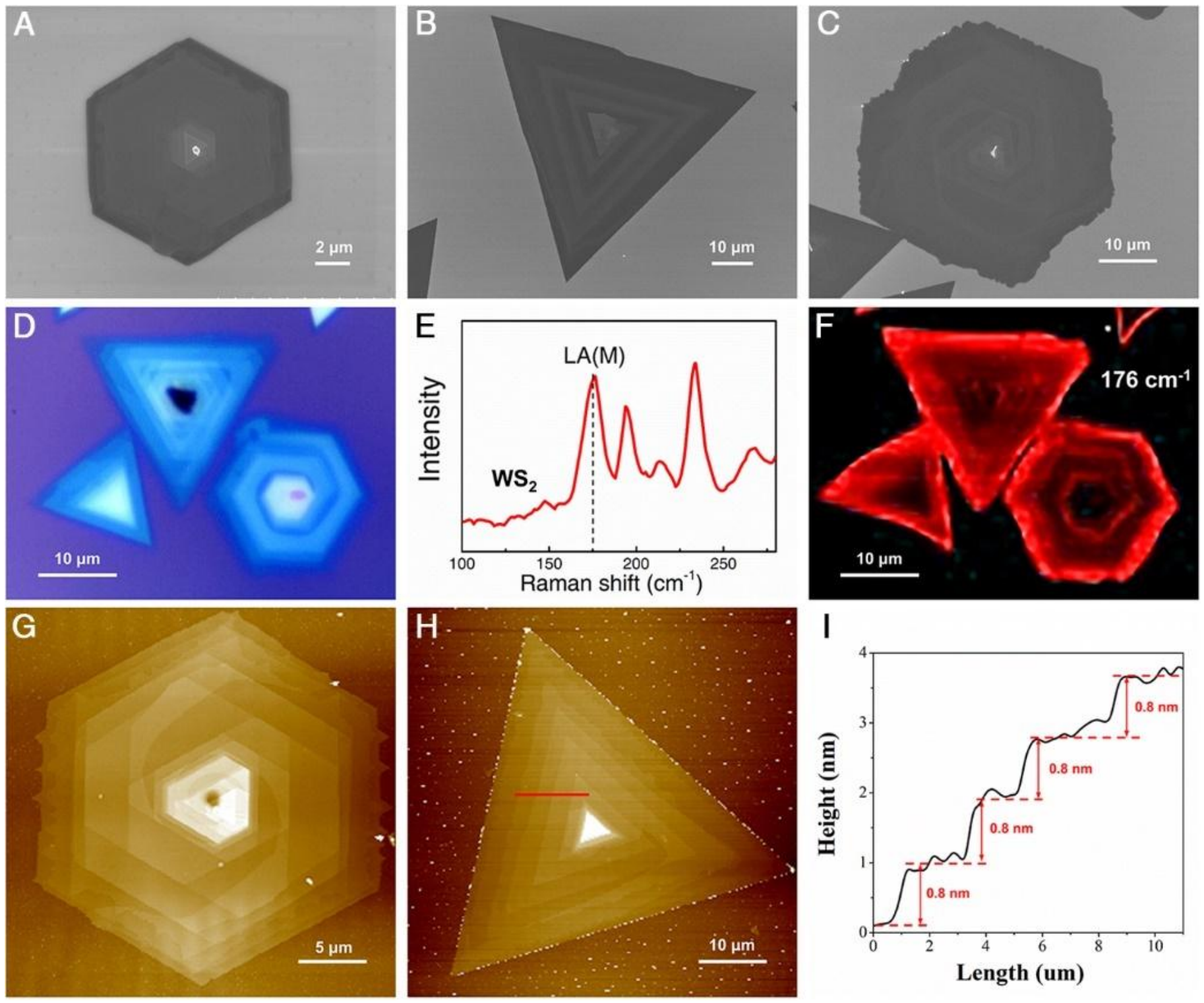

Fig. S2. Characterization of 2D spirally grown WS_2. (**A** to **C**) SEM images of three representative spiral growths of 2D WS_2 with different morphologies. (**D**) Optical microscope image of multiple spirally grown WS_2 samples. (**E**) Raman spectra of spirally grown WS_2. (**F**) Spatially resolved Raman mapping of multiple spirally grown WS_2 samples, showing a clear spiral growth pattern with darker central regions compared to the edges for each sample. (**G** and **H**) AFM image of two spirally grown WS_2. (**I**) Height profile along the red solid line in (H). The fabricated spiral samples are characterized by rich morphological features, including the multiple screw dislocation structures evident in (G) and (H), thereby establishing a material foundation for in-depth investigation of their growth mechanism.

Notably, the Raman mapping of spirally grown WS_2 shows that the edge regions are brighter than the central region. This contrast is associated with the longitudinal acoustic mode at the M point in the Brillouin zone, *LA*(**M**) (~176 cm^{-1}), which is highly sensitive to defects(*39, 40*). The reduced intensity of the *LA*(**M**) mode in the central region indicates a lower defect density on the surface of the spirally grown WS_2 in this area.

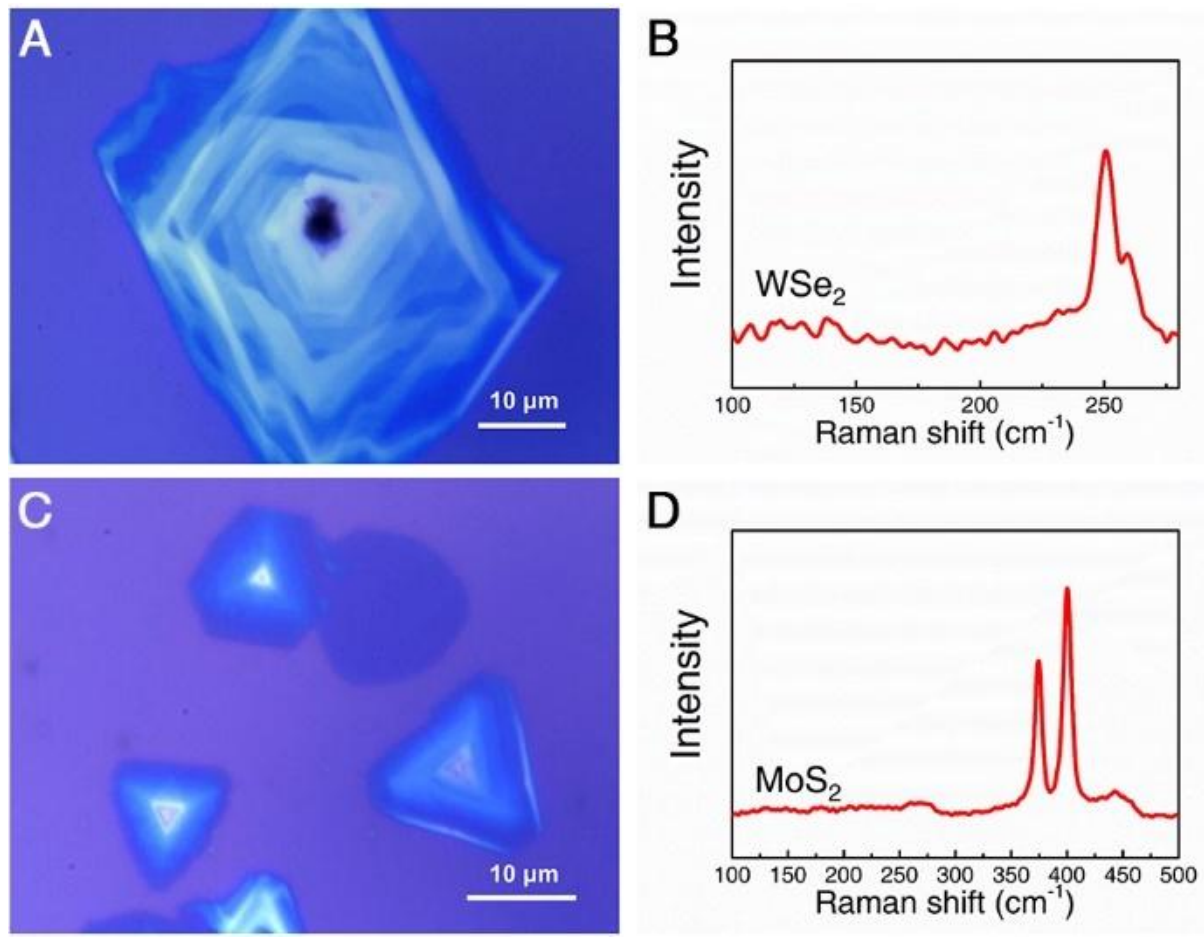

Fig. S3. Optical images and Raman spectra of spirally grown WSe_2 and MoS_2. (**A** and **B**) Optical image and Raman spectra of spirally grown 2D WSe_2 (*44*). (**C** and **D**) Optical image and Raman spectra of spirally grown 2D MoS_2 (*43*).

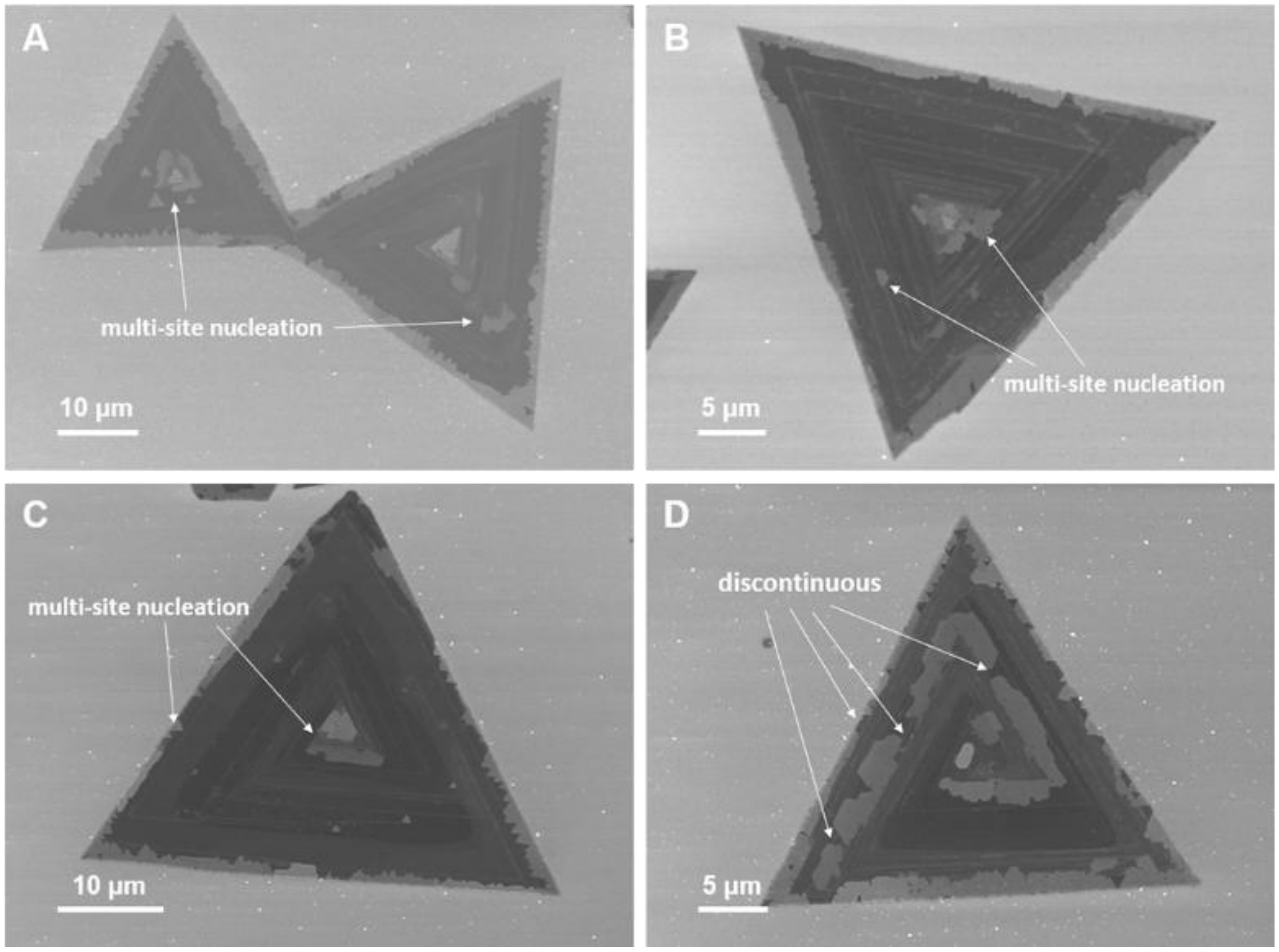

Fig. S4. Key evidence of multi-site nucleation. (**A** to **D**) SEM images of the early-stage growth of m-TMDs on s-TMDs show that m-TMDs nucleate at multiple discrete points along the edge of the s-TMDs sample and the spiral edge.

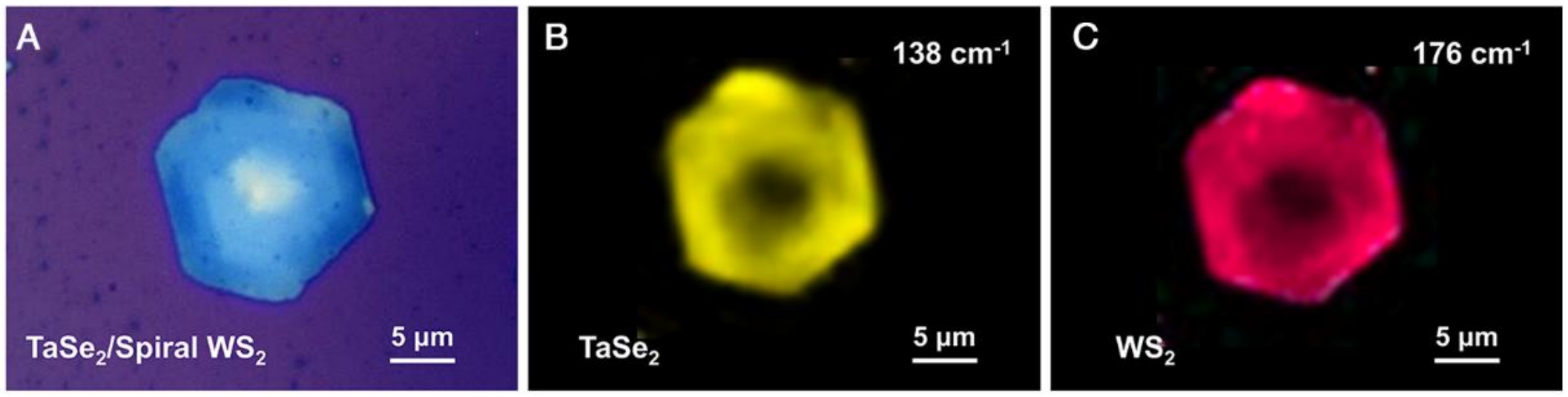

Fig. S5. Optical and Raman mapping characterization of 2D $TaSe_2$/Spiral WS_2 vdWHs. (**A**) Optical image of a 2D $TaSe_2$/Spiral WS_2 vdWHs with hexagonal morphology. (**B** and **C**) Spatially resolved Raman mapping images of the Raman modes at 138 cm^{-1} (E_{1g} of $TaSe_2$) and 176 cm^{-1} (*LA*(**M**) of WS_2), respectively (*39, 41*).

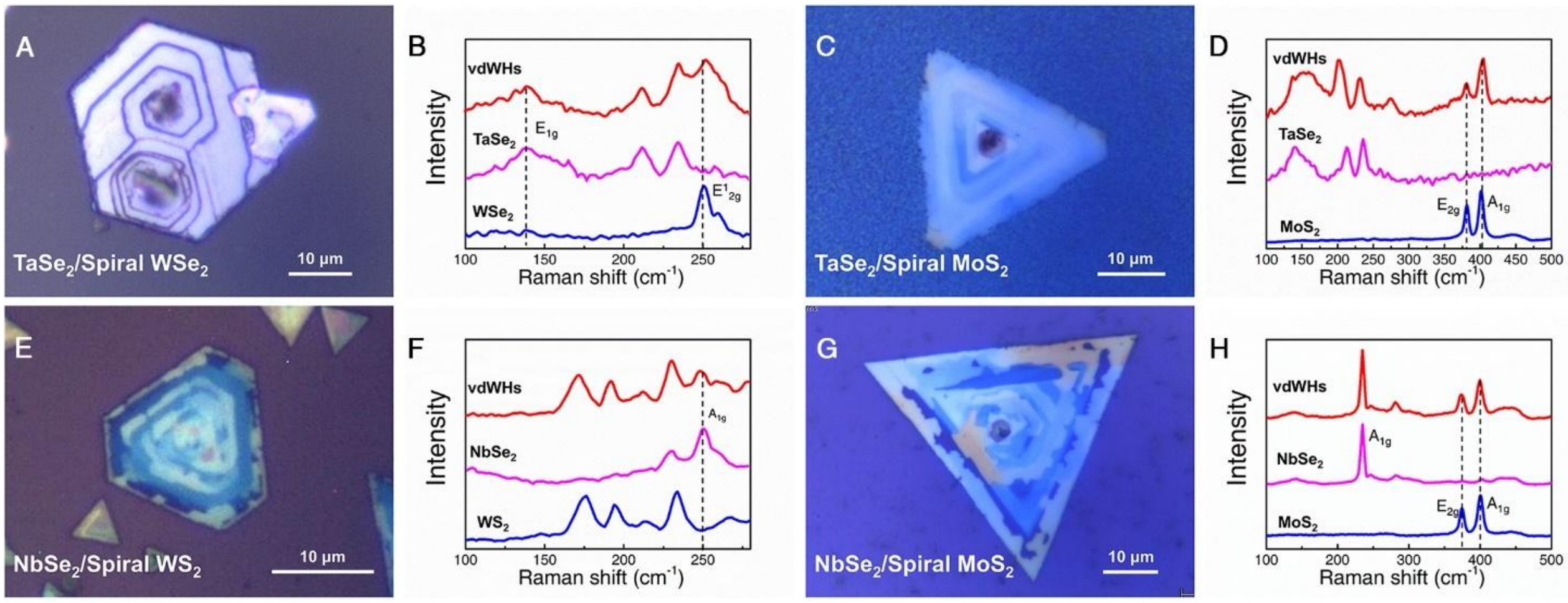

Fig. S6. Optical and Raman characterization of various m-TMDs/Spiral s-TMDs vdWHs. (**A** and **B**) Optical image and Raman spectra of a 2D $TaSe_2$/Spiral WSe_2 vdWHs (*41, 44*). (**C** and **D**) Optical image and Raman spectra of a 2D $TaSe_2$/Spiral MoS_2 vdWHs (*41, 43*). (**E** and **F**) Optical image and Raman spectra of a 2D $NbSe_2$/Spiral WS_2 vdWHs (*39, 42*). (**G** and **H**) Optical image and Raman spectra of a 2D $NbSe_2$/Spiral MoS_2 vdWHs (*42, 43*).

In our experiments, we observed that compared to $NbSe_2$, $TaSe_2$ exhibits a higher in-plane growth rate and more complete coverage on spirally grown s-TMDs templates. This difference may originate from their distinct growth kinetics, which consequently affects the final morphology of the epitaxial overlayer.

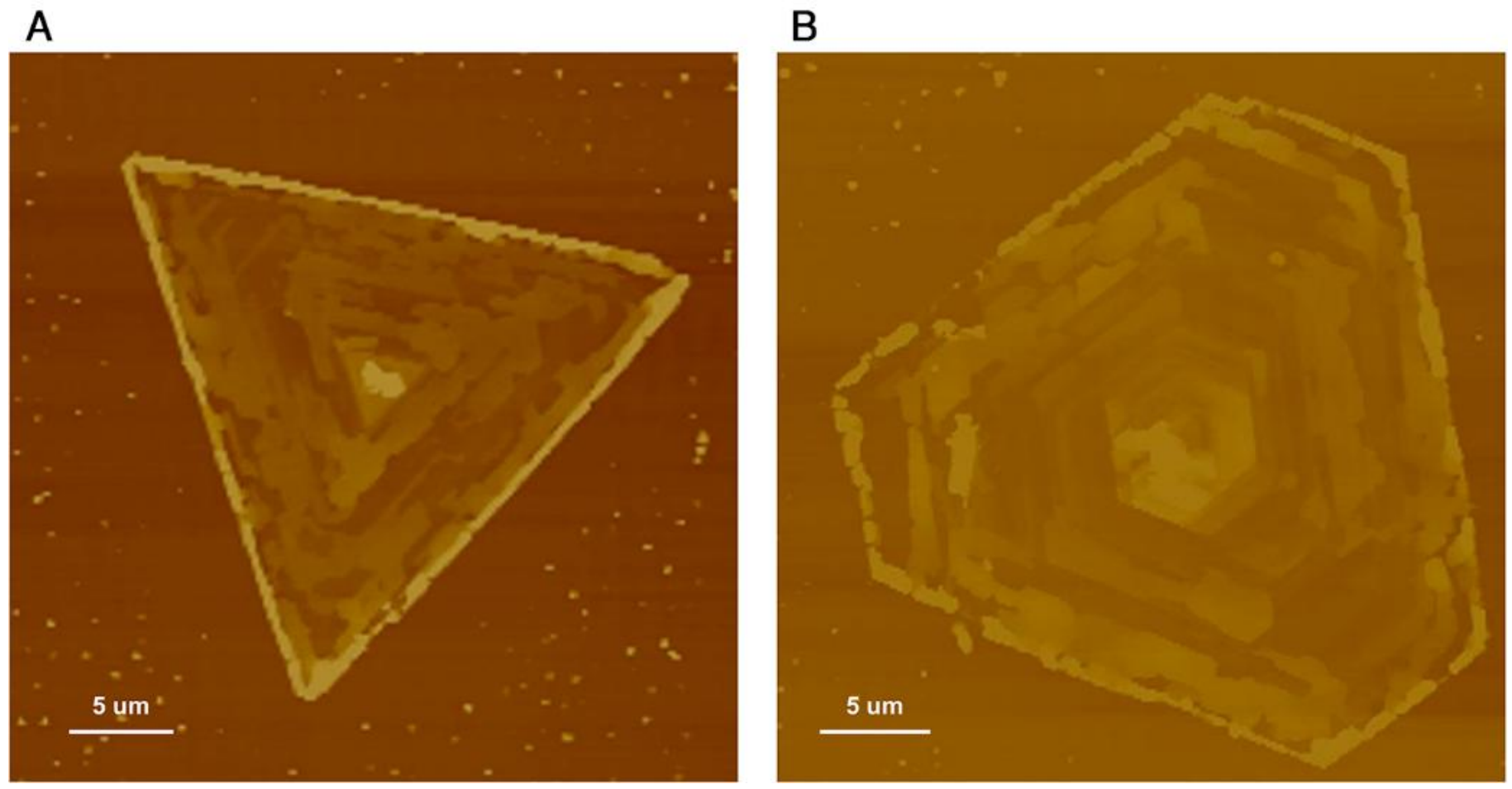

Fig. S7. AFM characterization of 2D $NbSe_2$/spiral WS_2 vdWHs. (**A** and **B**) AFM images of the 2D $NbSe_2$/Spiral WS_2 vdWHs with triangular (A) and hexagonal (B) morphology, respectively. The AFM images reveal that the epitaxial growth of $NbSe_2$ is discontinuous and does not result in complete coverage of the s-TMDs.

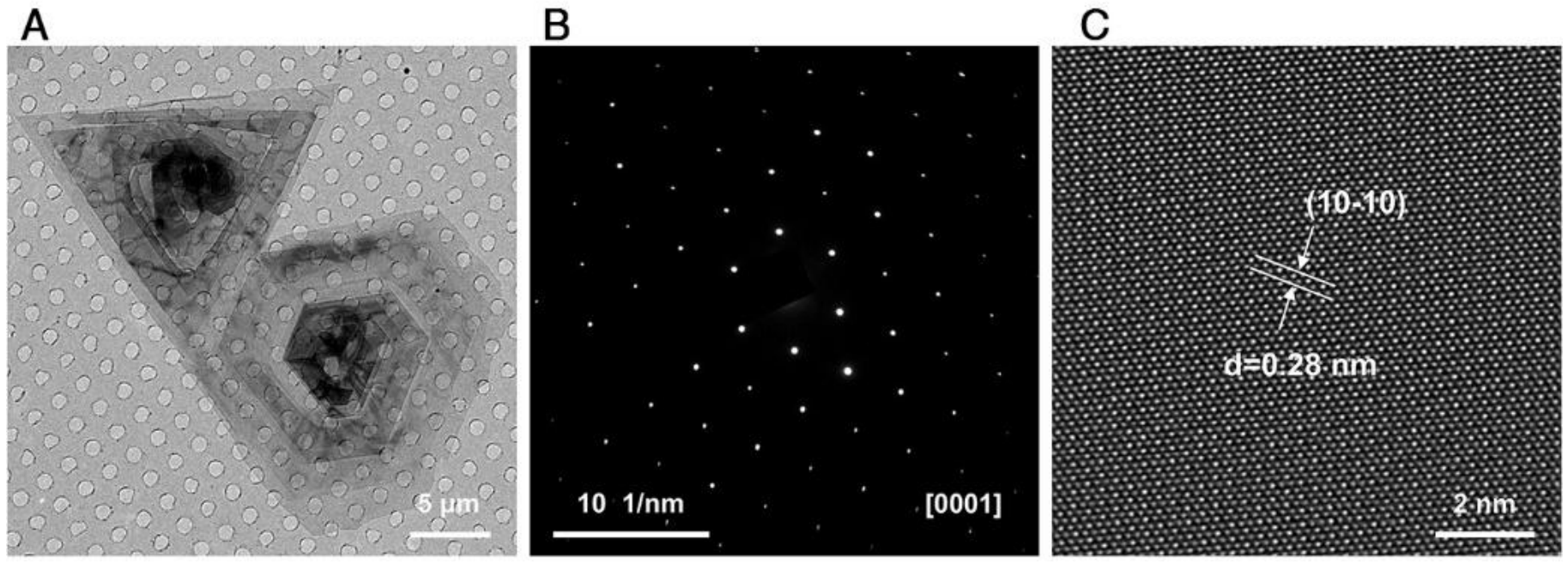

Fig. S8. Planar-view STEM characterization of spirally grown 2D TMDs. (**A**) Low-magnification HAADF-STEM image of spirally grown 2D WS_2 with hexagonal and triangular morphology. (**B** and **C**) SAED pattern (B) and atomic resolution HAADF-STEM image (C) of samples in (A).

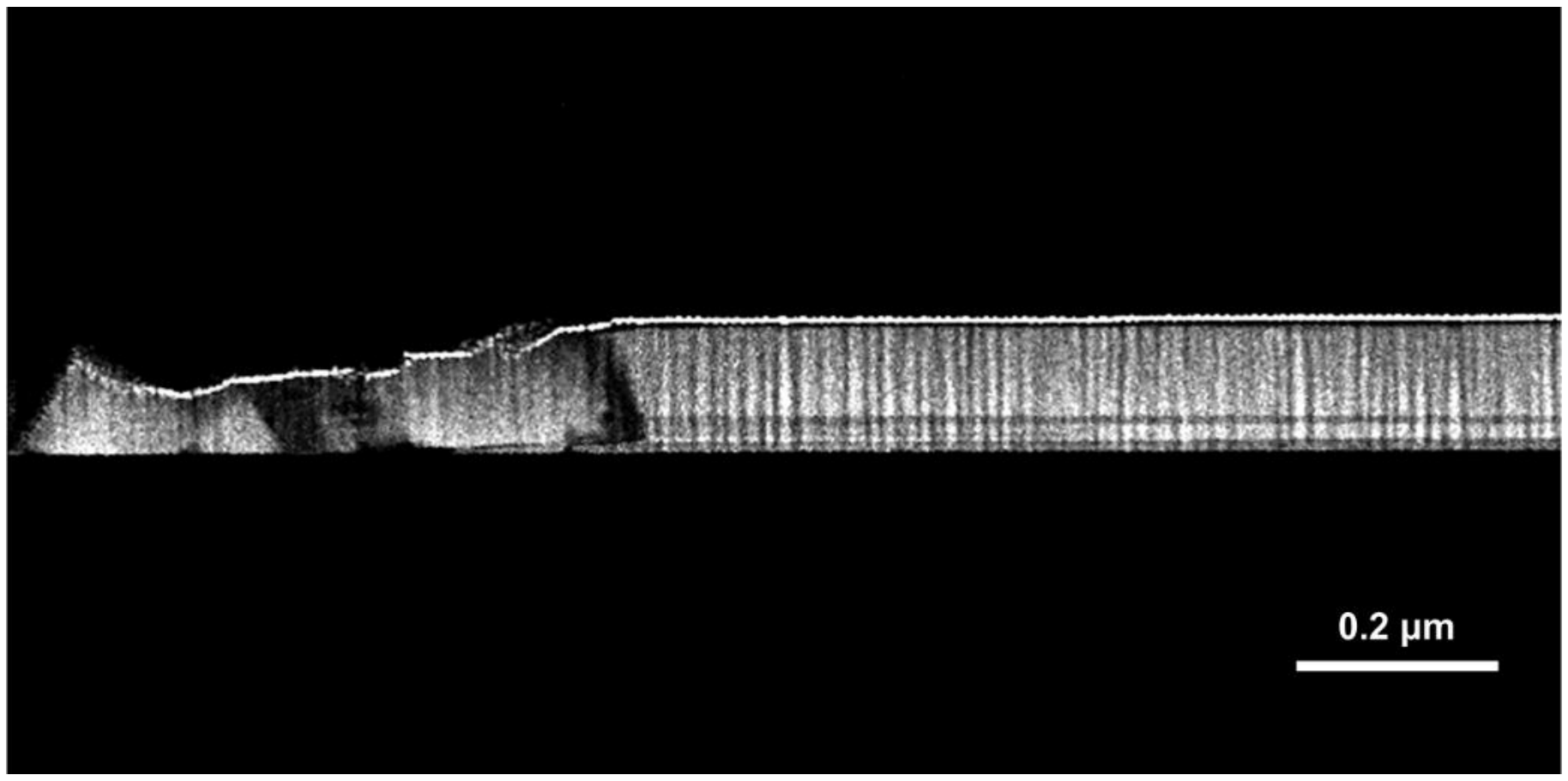

Fig. S9. Cross-sectional STEM observation of 2D $TaSe_2$/Spiral WS_2 vdWHs. The low-magnification HAADF-STEM image of a cross-sectional sample of $TaSe_2$/Spiral WS_2 vdWH reveals that the sample was extracted from the edge of the spiral vdWH. The edge region exhibits a simpler growth morphology, offering valuable insight into the fundamental growth behavior of the material on the substrate.

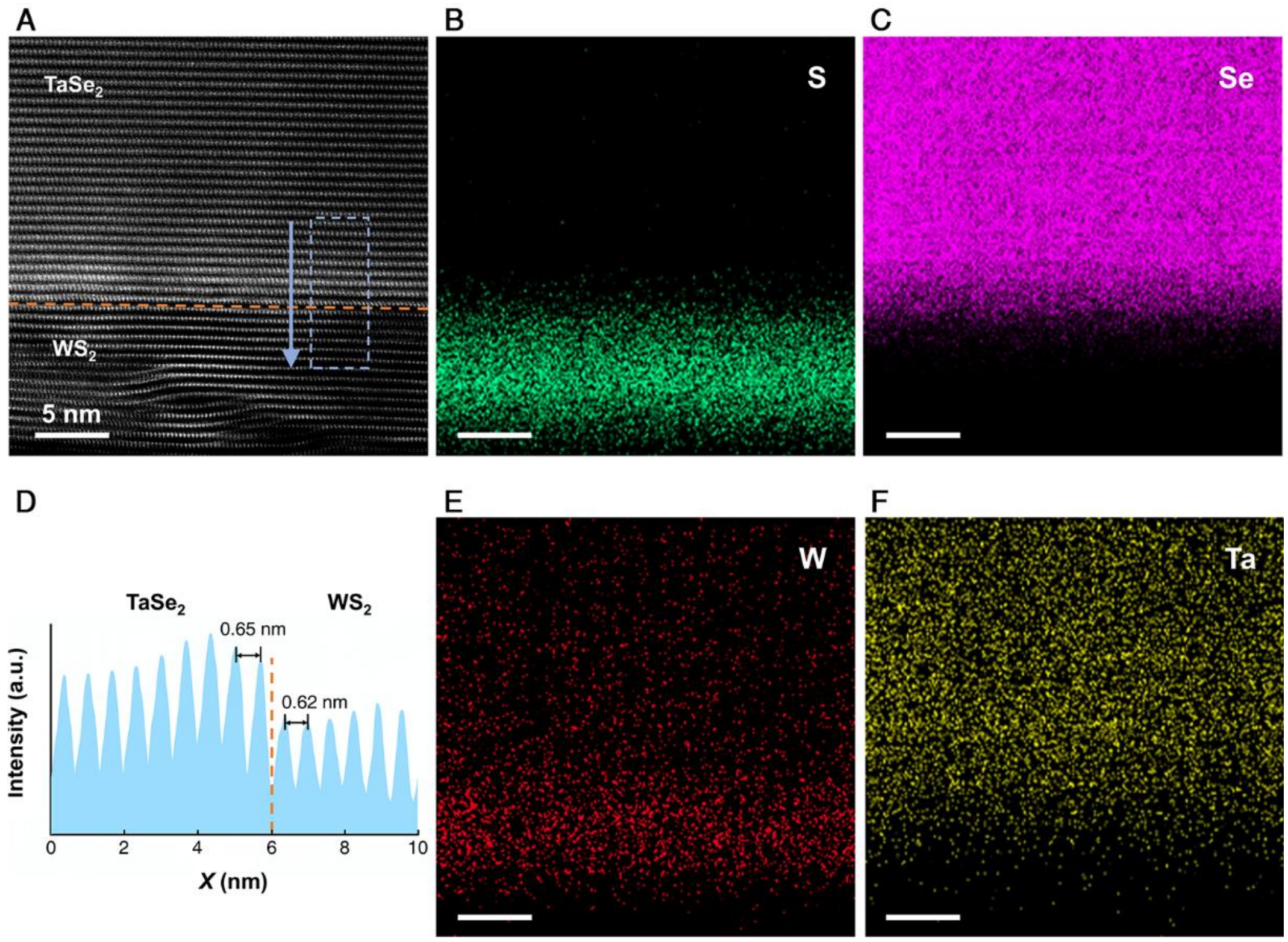

Fig. S10. Cross-sectional analysis of $TaSe_2$/Spiral WS_2 vdWHs interface. (A) HAADF-STEM image of a cross-sectional sample of the $TaSe_2$/Spiral WS_2 vdWH (adapted from Fig. 3E). (**B, C, E,** and **F**) Corresponding EDS elemental maps for S, Se, W, and Ta, respectively. Scale bars: 5 nm. (**D**) Intensity profile of the blue dashed area in (A). The orange dashed line indicates the heterostructure interface.

HAADF-STEM imaging, also known as *Z*-contrast imaging, exhibits signal intensity approximately proportional to the square of the atomic number (*73*). Given that the atomic number of tungsten (W, Z = 74) is higher than that of tantalum (Ta, Z = 73), in defect-free regions, the underlying WS_2 layer is expected to display stronger HAADF contrast than the overlying $TaSe_2$ layer. However, notable intensity inhomogeneity is observed within the WS_2 layer, which originates from its growth mechanism dominated by MSDD processes. At the dislocation cores, atomic columns undergo severe distortion or even rupture, leading to a reduction in local effective thickness and consequently a decrease in high-angle scattering efficiency, manifesting as localized dark contrast in the images. Simultaneously, the $TaSe_2$ layer grown epitaxially on the WS_2 surface follows a LbL growth mode. Modulated by the strain field induced by the underlying WS_2 surface structure, the epitaxial layer exhibits alternating bright and dark vertical stripes distributed throughout the cross-sectional sample.

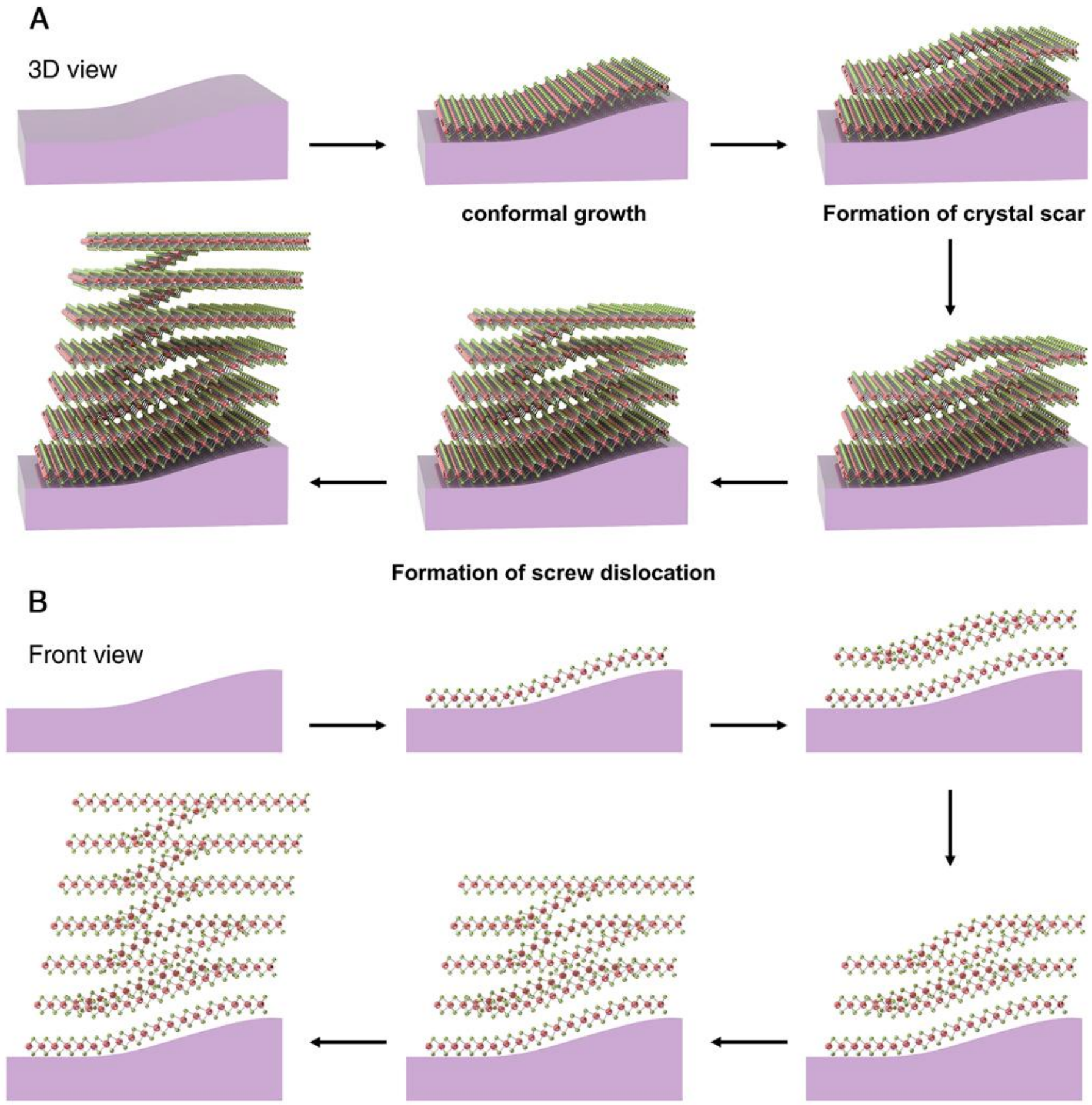

Fig. S11. Schematic depiction of vertical growth of 2D materials on a curved surface. (**A** and **B**) Schematic illustration of 2D material vertical growth on a curved surface, depicted in (A) 3D and (B) front views. Initially, the first layer grows conformally along the substrate surface, followed by LbL growth featuring topological scars. This is eventually followed by SDD growth. Created with Blender.

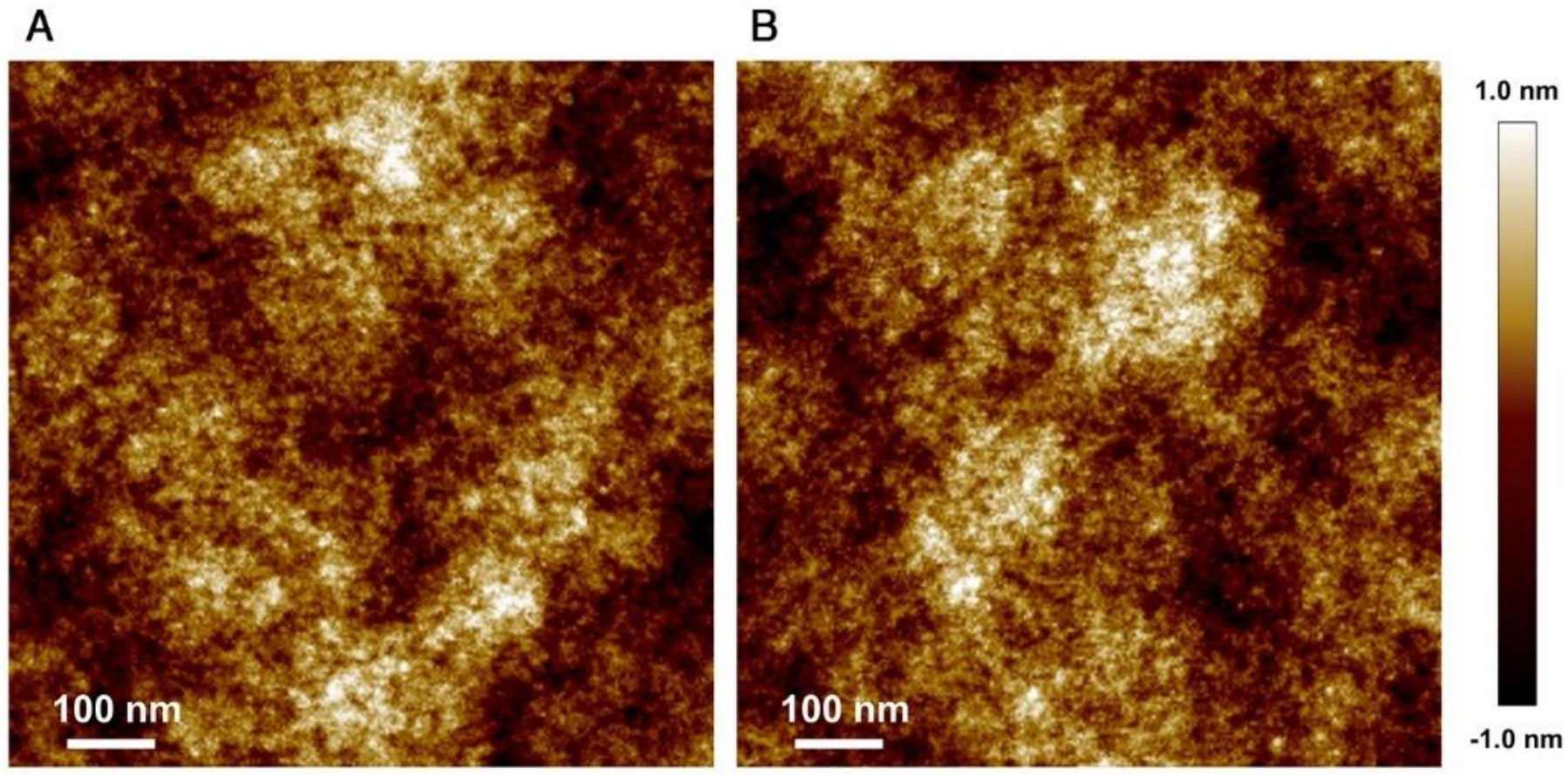

Fig. S12. AFM characterization of substrate. (**A** and **B**) AFM images of SiO_2/Si substrates (A) before and (B) after piranha solution treatment. The images confirm the presence of nanoscale undulations with sub-nanometer roughness. Measured roughness values are Ra = 0.264 nm and Rq = 0.329 nm (before treatment) and Ra = 0.268 nm and Rq = 0.337 nm (after treatment).

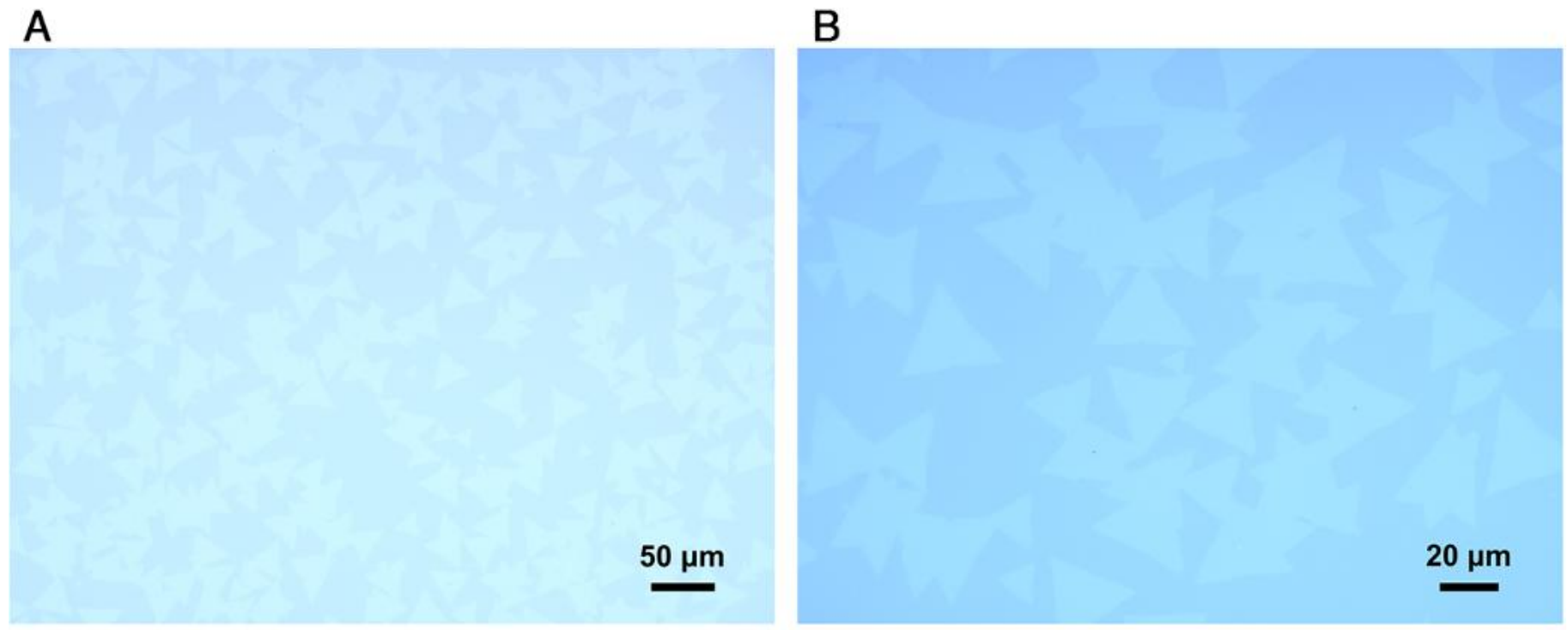

Fig. S13. Optical characterization of TMDs grown on sapphire substrate. (**A** and **B**) Optical microscopy images at different magnifications showing the growth of 2D WS_2 on an atomically flat sapphire substrate. Compared to the silica substrate, the 2D WS_2 grown under identical experimental conditions on the atomically flat sapphire substrate exhibits a flake-like morphology, with no spiral growth observed.

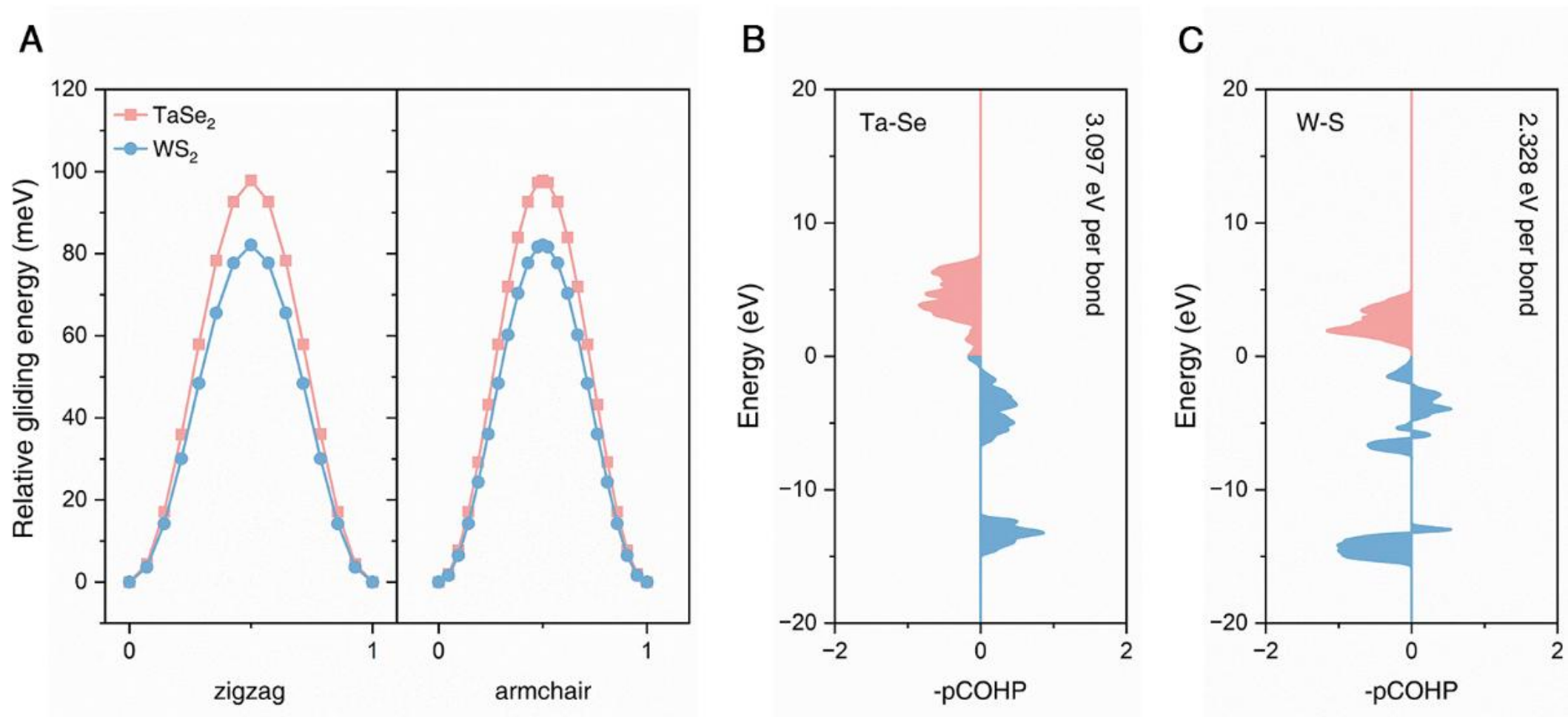

Fig. S14. First-principles calculations. (**A**) Gliding energy barriers along a period of zigzag $\langle 1\bar{2}10 \rangle$ and armchair $\langle 10\bar{1}0 \rangle$ directions for 3R phases of WS_2 and $TaSe_2$. (**B** and **C**) Partial COHP for intralayer bonding of Ta-Se (B) and W-S (C). The integration of COHP (in eV per bond) for the blue part.

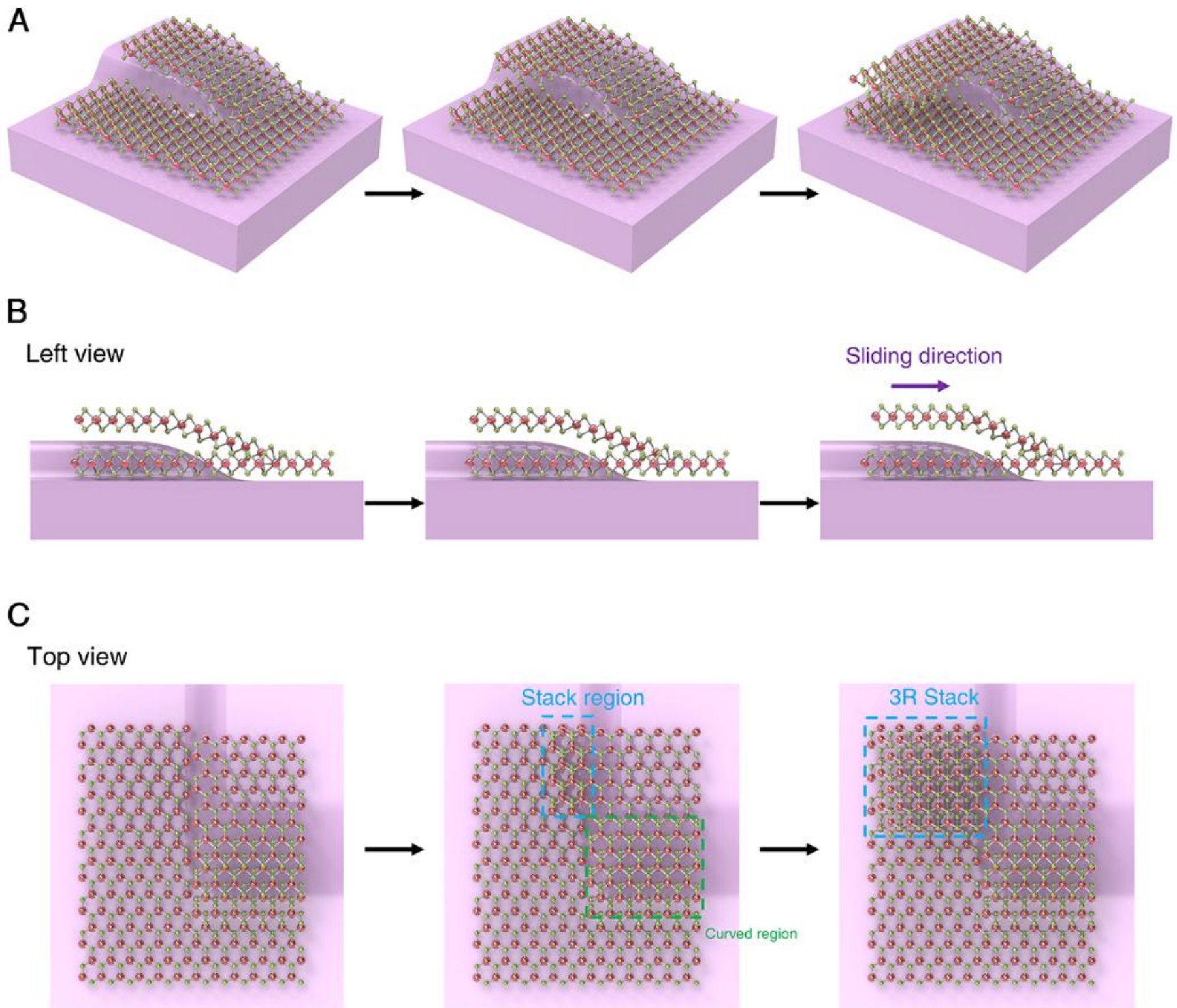

Fig. S15. Schematic representation of stacking transition in 2D TMDs. (**A** to **C**) Schematic diagrams showing the transition from random stacking to 3R stacking in 2D materials, presented in (A) 3D view, (B) left view, and (C) top view. Initially, 2D materials grow with random stacking, and as the stacked layers expand, the random stacking eventually transitions to 3R due to the competition between slip and strain energies (generated by bending). Created with Blender.

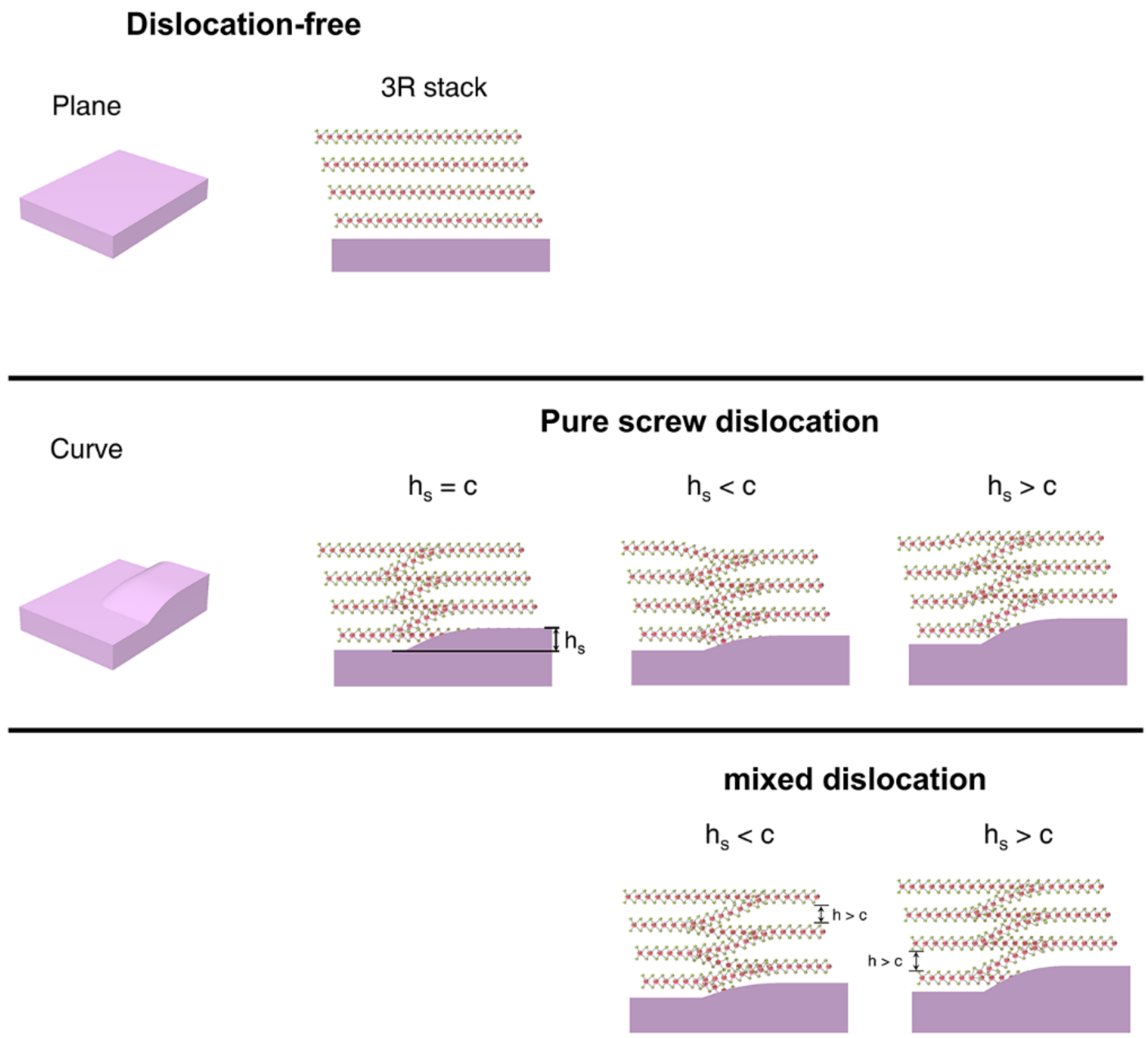

Fig. S16. Schematic diagram illustrating the classification of individual SDD growth in 2D materials. From top to bottom: 3R stacking without dislocations, pure screw dislocation growth, and mixed dislocation growth, respectively. The change in layer spacing is characteristic of mixed dislocation. Created with Blender.

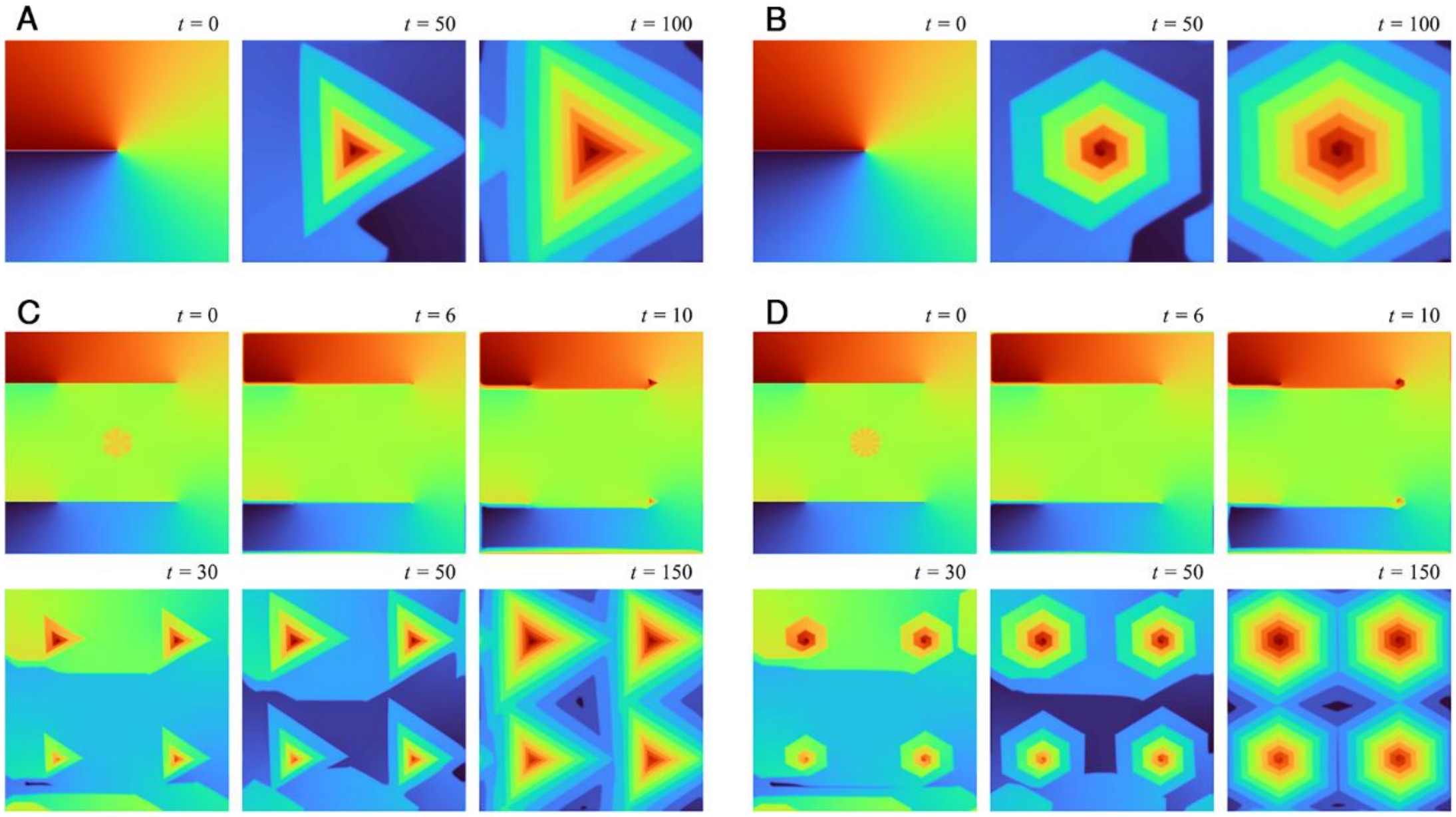

Fig. S17. Phase-field simulations of MSDD growth. (**A** and **B**) Phase-field profiles ϕ of spiral growth from a single screw dislocation at different dimensionless times, showing triangular (A) and hexagonal (B) morphologies. (**C** and **D**) Phase-field profiles ϕ of spiral growth from four screw dislocation at different dimensionless times, showing triangular (C) and hexagonal (D) morphologies. The growth process of MSDD undergoes a complete evolution, from initial nucleation (t=0) and conformal in-plane expansion (t=6) to curvature-induced screw dislocation formation (t=10), and finally results in the development of a multi-screw dislocation structure (t=30, 50, and 150). Color represents height, increasing from blue (low) to red (high). This color scale applies individually to each image, showing relative variation within its own structure, and does not serve as a universal scale for comparing absolute heights between different structures.

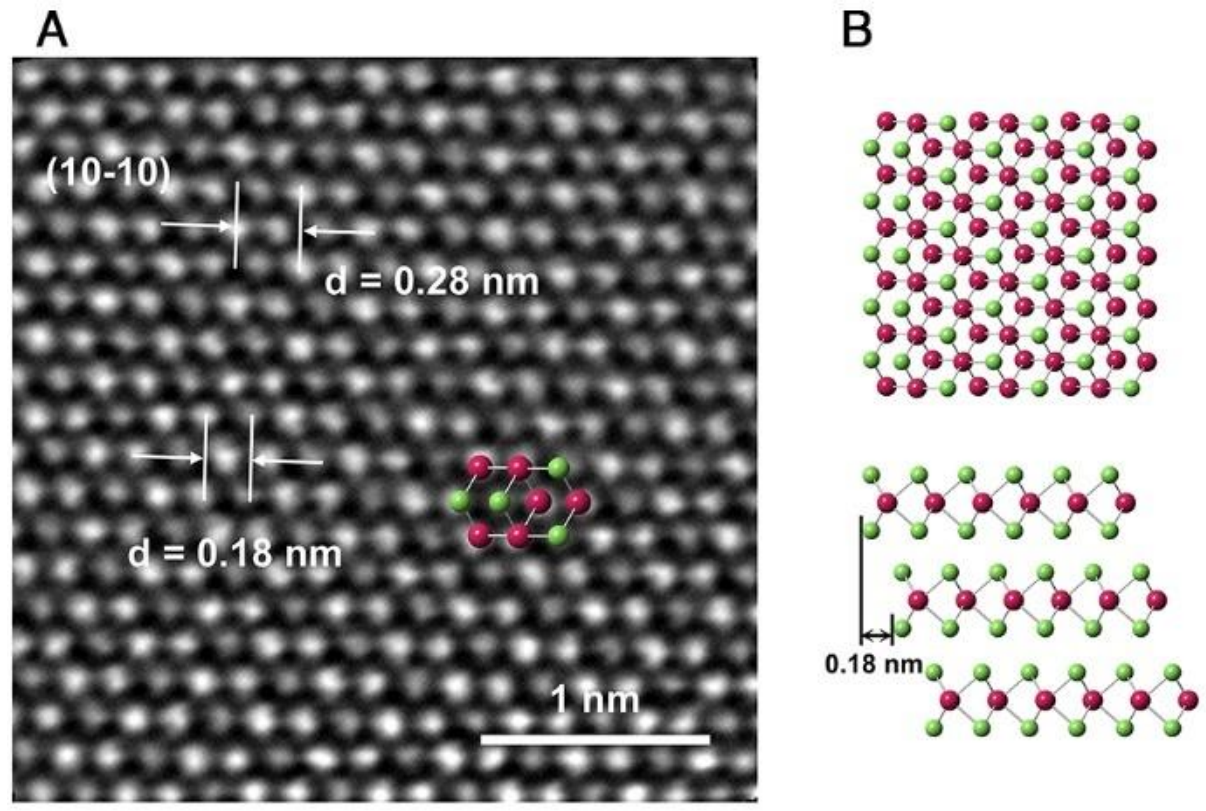

Fig. S18. Atomic-scale structural characterization of 3R-stacked TMDs. (**A**) Atomic resolution HAADF-STEM image of 3R stacked WS_2. **(B)** Schematic diagram of 3R stacking.

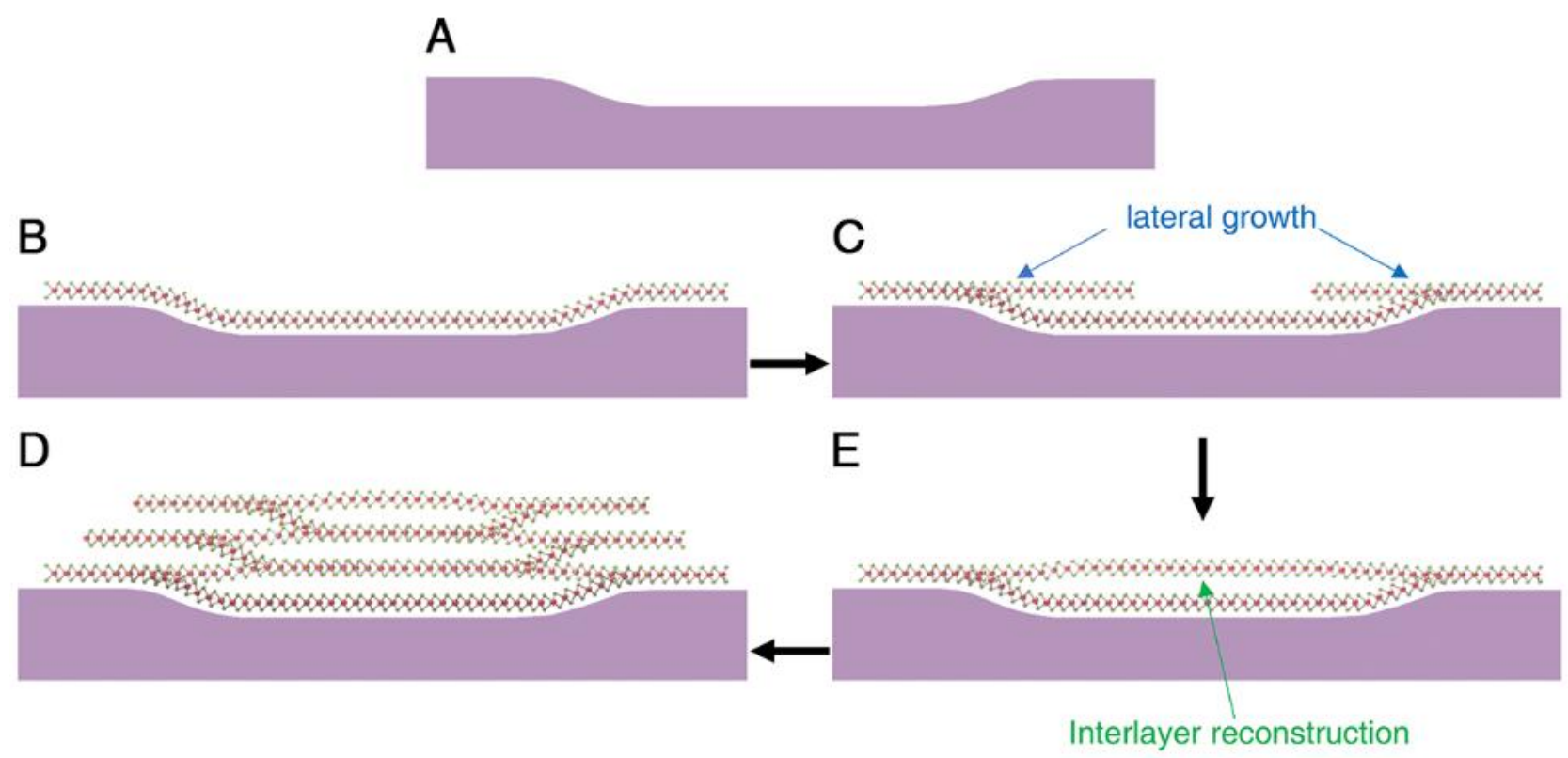

Fig. S19. **Schematic representation of interlayer reconstruction.** (**A**) Substrate used for growth. (**B**) Conformal growth of 2D material on the substrate surface. (**C**) Curved surface leading to screw dislocation formation and lateral growth. (**D** and **E**) Interlayer reconstruction (slipping and variations in interlayer distance) resulting from layer merging between screw dislocations. Created with Blender.

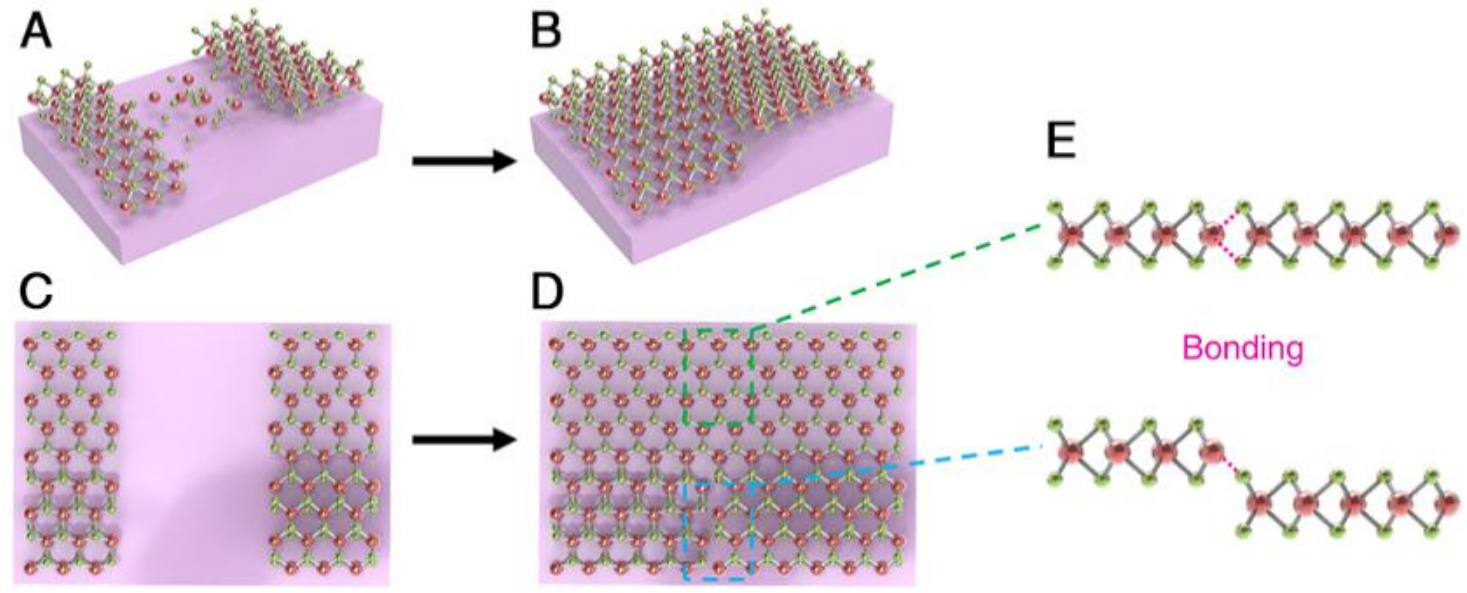

Fig. S20. Schematic diagram illustrating the formation of a topological scar structure. (**A** and **B**) A small out-of-plane displacement between adjacent layers leads to their merging. (**C** to **E**) The out-of-plane displacements are larger but insufficient to induce screw dislocation growth, leading to the bonding of W elements only to the upper S elements, thereby forming a topological scar structure. Created with Blender.

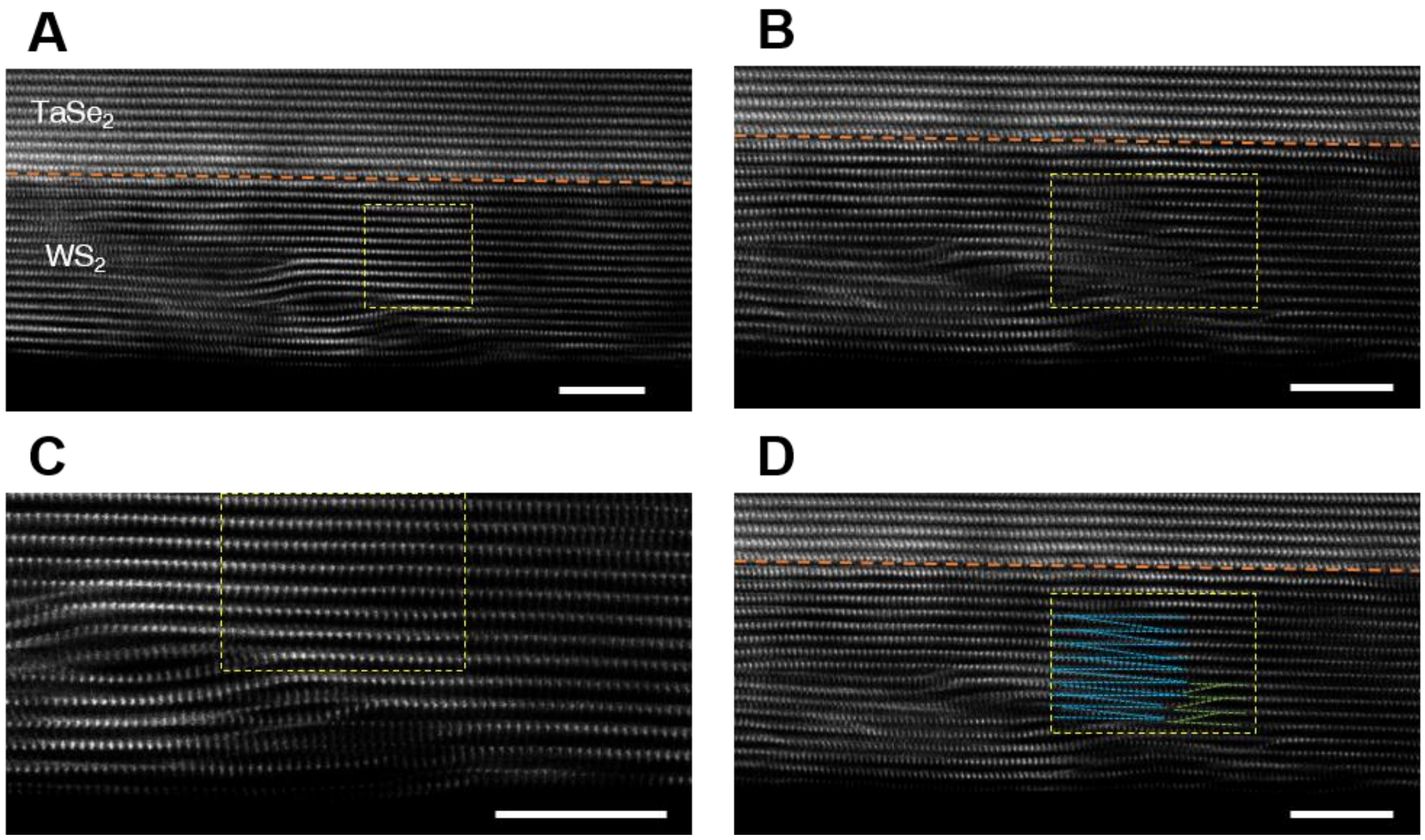

Fig. S21. Cross-sectional STEM characterization of dislocations within $TaSe_2$/Spiral WS_2 vdWHs. (**A** to **C**) HAADF-STEM images of the same characteristic region (yellow dashed box) acquired at different magnifications. The orange dashed line marks the position of the heterostructure interface. (**D**) The same image as (B), with dislocations marked.

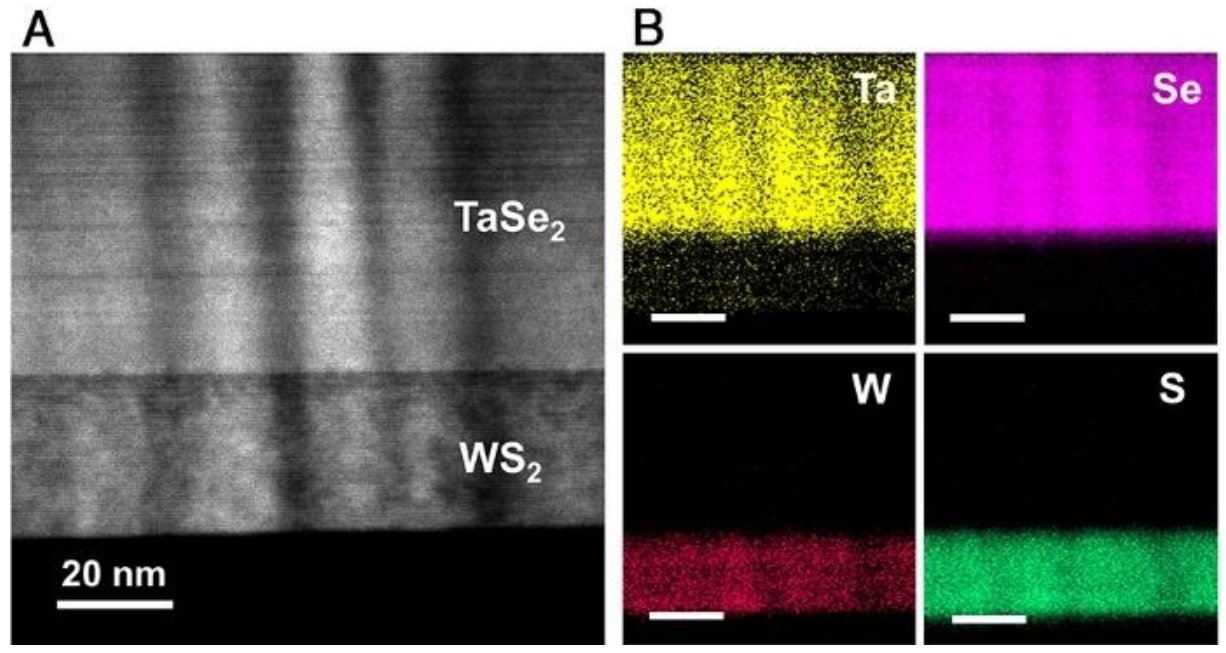

Fig. S22. Cross-sectional STEM characterization of $TaSe_2$/Spiral WS_2. (**A**) HAADF-STEM image of a cross-sectional sample of $TaSe_2$/Spiral WS_2 vdWH. (**B**) The EDS elemental maps of the 2D $TaSe_2$/Spiral WS_2 vdWH. Scale bars: 25 nm.

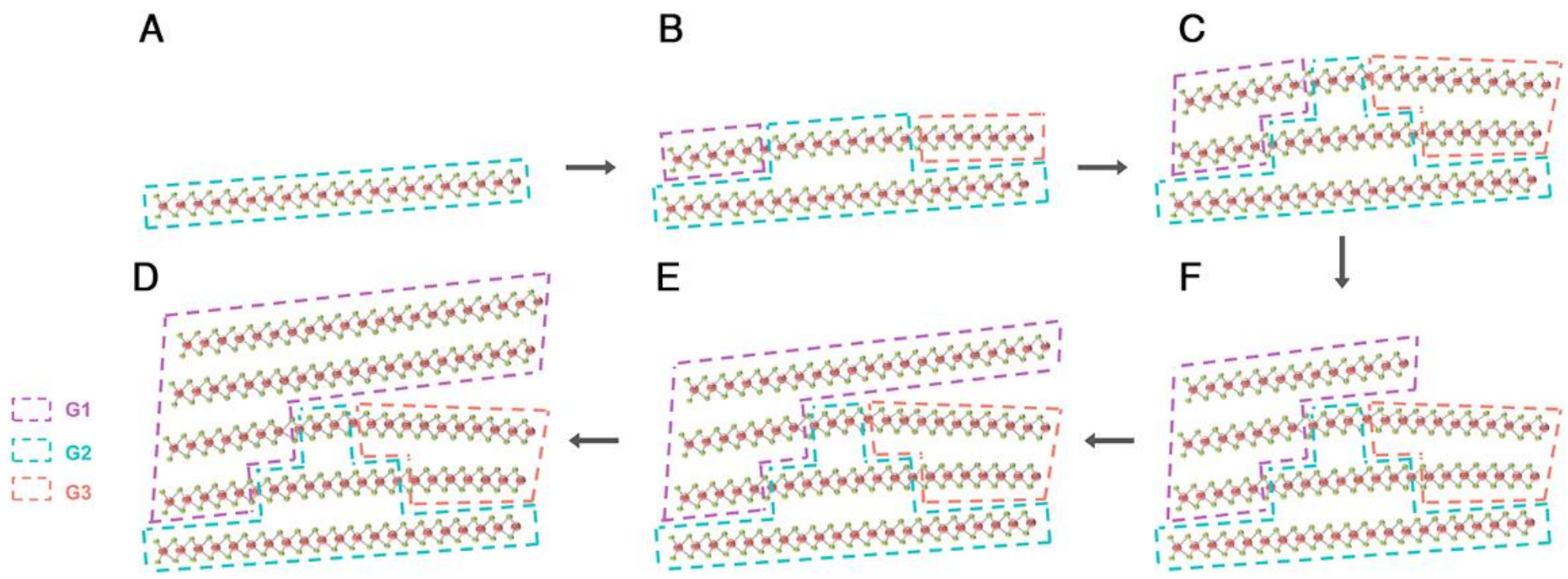

Fig. S23. **Schematic diagram of the competitive growth mechanism.** (**A**) The initial layer is a G2 grain. (**B** and **C**) SDD growth dominates vertical growth and the width of G2 grain decreases. (**D** and **E**) The G1 grain front grows laterally and forms a LAGB on the G3 grain surface. (**F**) The curvature affects the growth rate of the SDD so that the lateral growth of the G1 grains dominates the subsequent growth.

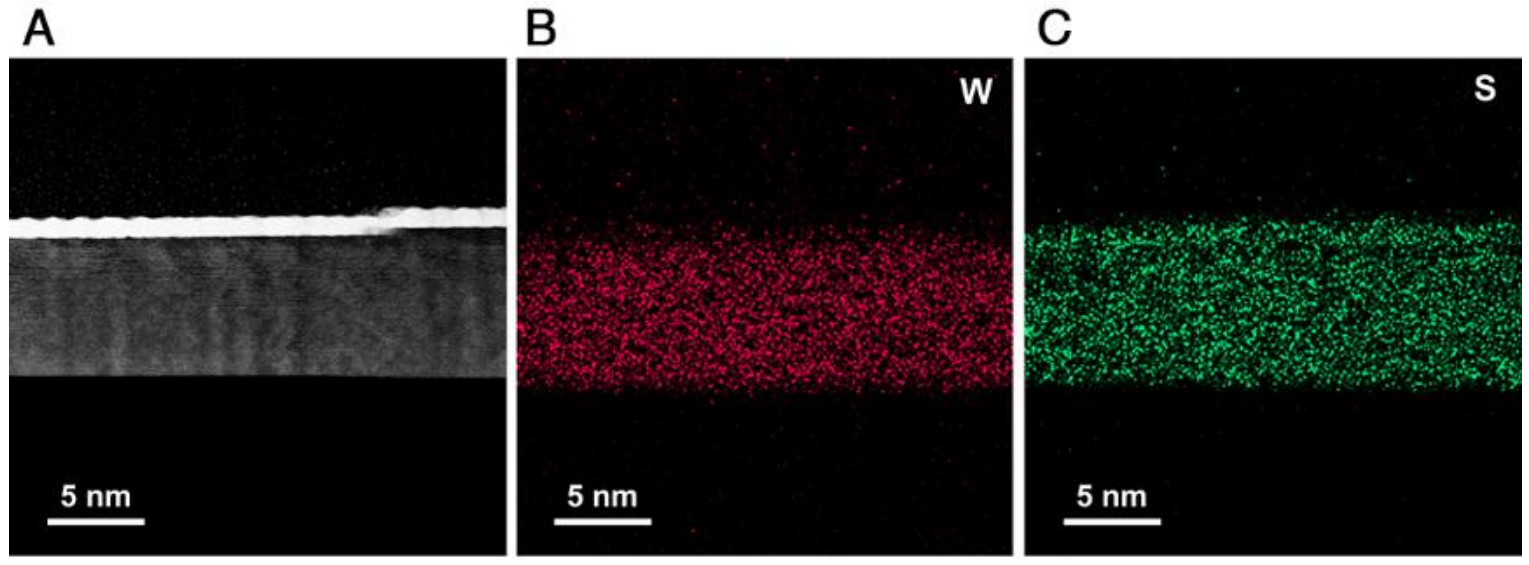

Fig. S24. Cross-sectional STEM characterization of spirally grown WS_2. (**A**) Low-magnification HAADF-STEM image of a cross-sectional sample of spirally grown WS_2. (**B** and **C**) The EDS elemental maps of spirally grown WS_2.

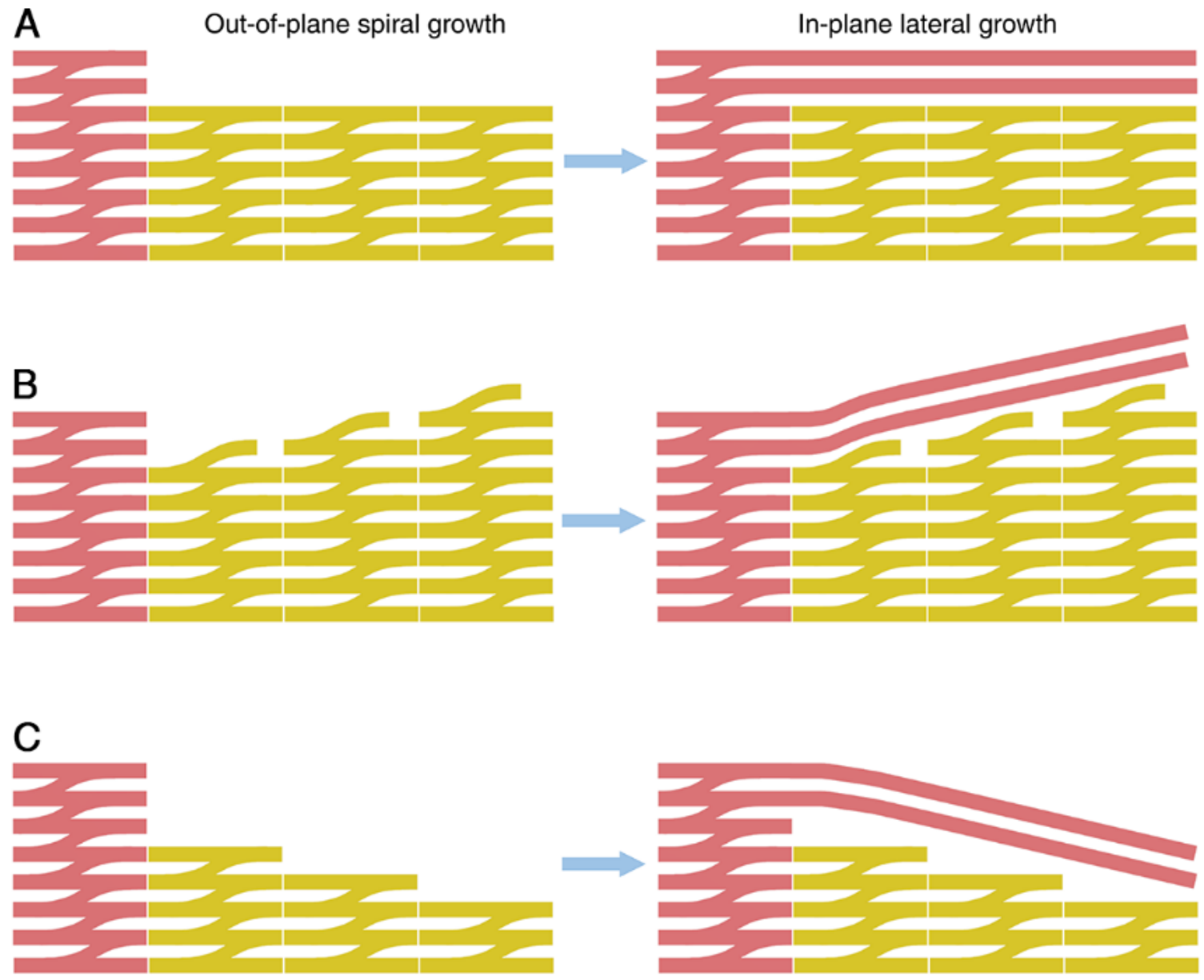

Fig. S25. Schematic illustration of dislocation annihilation. (**A** to **C**) A growth-advantaged dislocation domain expands laterally to cover adjacent slower-growing domains, thereby cutting off their material supply and leading to growth termination. Such competitive growth and coverage directly lead to the formation of structures observed in experiments, including LbL growth (A), LAGB (B), and crack (C).

Note S1. The Fundamental Limitations of Cross-Sectional STEM Characterization

Our HAADF-STEM images are 2D projections through approximately 20 nm thick lamellae, while the screw dislocation cores are only about 5 nm in size (*25, 27*). Several fundamental factors limit the information that can be extracted from such characterization regarding multiple screw dislocations:

Projection effect: The image integrates information through the entire lamella thickness, making it impossible to determine the exact 3D position of dislocation cores. Particularly in multi-dislocation systems, dislocations that appear to meet or intersect in the image may simply be spatial projections of cores at different depths overlapping along the viewing direction, rather than true physical contact or interaction.

Sign indeterminacy: The sign of screw dislocations cannot be determined from a single cross-sectional image. This information is essential for predicting whether two screw dislocations will annihilate upon meeting, yet it remains fundamentally inaccessible from 2D projection imaging.

Complexities in multi-dislocation imaging: In STEM characterization, deterministic imaging of screw dislocations has always been challenging due to the complexity of their structures. The image quality is highly dependent on the microscope's operating conditions, such as focus, sample orientation, and astigmatism correction (*22*). This indicates that for a single screw dislocation, even slight variations in the orientation of the connecting layers can make imaging difficult. This is even more pronounced for multiple screw dislocations, as the crystallographic orientations of individual dislocations are often not perfectly aligned. Consequently, it is challenging to simultaneously characterize all screw dislocations with optimal contrast in a single STEM image; instead, analysis typically focuses on specific regions of interest.

Note S2. The Intrinsic Complexity of Multi-Dislocation Systems

Multi-screw dislocation growth inherently involves complex interactions that are not present in single-dislocation systems:

Stacking reconstruction: When adjacent screw dislocation growth fronts merge, localized lattice stresses are induced, which drive interlayer stacking reconstruction, manifesting as the anomalous stacking phases we observe.

Dislocation meandering: Dislocation lines are not perfectly perpendicular to the atomic planes; they undergo continuous spatial evolution during growth, often exhibiting complex meandering patterns. This occurs because screw dislocations induced by substrate undulations typically adopt a 3R stacking, in which the dislocation lines are inherently inclined, and the specific inclination direction of each individual screw dislocation is random. During growth, the stress generated by multiple screw dislocations can induce lattice tension, compression, or variations in interlayer spacing, thereby introducing edge dislocations. This transforms screw dislocations into mixed dislocations, making the evolution of dislocation lines even more unpredictable. Consequently, dislocations that originate from substrate undulations in the underlying layers may deviate from the intended imaging region during subsequent growth—or even extend entirely out of the section—creating a false impression of dislocation termination or layer-by-layer growth. This is particularly relevant to the “layer-by-layer” regions pointed out by the reviewer.

Merging of opposite-sign dislocations: Dislocations with opposite signs attract each other and can merge, leading to the annihilation of spiral growth steps.

Competitive growth for precursor supply: Dislocations compete for limited precursor supply. Those with growth advantages can expand laterally and cover slower-growing neighbors, cutting off their material supply and leading to growth termination. This “coverage mechanism” is central to our MSDD framework and explains features such as cavity formation.

Interaction-induced distortions: When multiple dislocations interact, the resulting lattice distortions and orientation changes further degrade image quality, making precise core localization even more difficult. The strain fields of neighboring dislocations can cause local tilting that moves the crystal away from zone axis conditions, reducing contrast and obscuring details.

REFERENCES

1. F. C. Frank, W. T. Read, Multiplication processes for slow moving dislocations. *Phys. Rev.* **79**, 722–723 (1950).

2. W. K. Burton, N. Cabrera, F. C. Frank, The growth of crystals and the equilibrium structure of their surfaces. *Philos. Trans. R. Soc. A* **243**, 299–358 (1951).

3. W. K. Burton, N. Cabrera, F. C. Frank, Role of dislocations in crystal growth. *Nature* **163**, 398–399 (1949).

4. F. C. Frank, The influence of dislocations on crystal growth. *Discuss. Faraday Soc.* **5**, 48–54 (1949).

5. J. D. Eshelby, The twist in a crystal whisker containing a dislocation. *Philos. Mag.* **3**, 440–447 (1958).

6. J. D. Eshelby, Screw dislocations in thin rods. *J. Appl. Phys.* **24**, 176–179 (1953).

7. S. A. Morin, M. J. Bierman, J. Tong, S. Jin, Mechanism and kinetics of spontaneous nanotube growth driven by screw dislocations. *Science* **328**, 476–480 (2010).

8. M. J. Bierman, Y. K. A. Lau, A. V. Kvit, A. L. Schmitt, S. Jin, Dislocation-driven nanowire growth and eshelby twist. *Science* **320**, 1060–1063 (2008).

9. J. Zhu, H. L. Peng, A. F. Marshall, D. M. Barnett, W. D. Nix, Y. Cui, Formation of chiral branched nanowires by the Eshelby Twist. *Nat. Nanotechnol.* **3**, 477–481 (2008).

10. Y. Liu, J. Wang, S. Kim, H. Sun, F. Yang, Z. Fang, N. Tamura, R. Zhang, X. Song, J. Wen, B. Z. Xu, M. Wang, S. Lin, Q. Yu, K. B. Tom, Y. Deng, J. Turner, E. Chan, D. Jin, R. O. Ritchie, A. M. Minor, D. C. Chrzan, M. C. Scott, J. Yao, Helical van der Waals crystals with discretized Eshelby twist. *Nature* **570**, 358–362 (2019).

11. T. H. Ly, J. Zhao, H. Kim, G. H. Han, H. Nam, Y. H. Lee, Vertically conductive MoS_2 spiral pyramid. *Adv. Mater.* **28**, 7723–7728 (2016).

12. M. J. Shearer, L. Samad, Y. Zhang, Y. Zhao, A. Puretzky, K. W. Eliceiri, J. C. Wright, R. J. Hamers, S. Jin, Complex and noncentrosymmetric stacking of layered metal dichalcogenide materials created by screw dislocations. *J. Am. Chem. Soc.* **139**, 3496–3504 (2017).

13. X. Fan, Y. Zhao, W. Zheng, H. Li, X. Wu, X. Hu, X. Zhang, X. Zhu, Q. Zhang, X. Wang, B. Yang, J. Chen, S. Jin, A. Pan, Controllable growth and formation mechanisms of dislocated WS_2 spirals. *Nano Lett.* **18**, 3885–3892 (2018).

14. Y. Zhao, C. Zhang, D. D. Kohler, J. M. Scheeler, J. C. Wright, P. M. Voyles, S. Jin, Supertwisted spirals of layered materials enabled by growth on non-Euclidean surfaces. *Science* **370**, 442–445 (2020).

15. Y. Nie, A. T. Barton, R. Addou, Y. Zheng, L. A. Walsh, S. M. Eichfeld, R. Yue, C. R. Cormier, C. Zhang, Q. Wang, C. Liang, J. A. Robinson, M. Kim, W. Vandenberghe, L. Colombo, P.-R. Cha, R. M. Wallace, C. L. Hinkle, K. Cho, Dislocation driven spiral and non-spiral growth in layered chalcogenides. *Nanoscale* **10**, 15023–15034 (2018).

16. Y. Zhao, X. Kong, M. J. Shearer, F. Ding, S. Jin, Chemical etching of screw dislocated transition metal dichalcogenides. *Nano Lett.* **21**, 7815–7822 (2021).

17. H. C. Lee, S. B. Jo, E. Lee, M. S. Yoo, H. H. Kim, S. K. Lee, W. H. Lee, K. Cho, Facet-mediated growth of high-quality monolayer graphene on arbitrarily rough copper surfaces. *Adv. Mater.* **28**, 2010–2017 (2016).

18. L. Guo, Z. Zhang, H. Sun, D. Dai, J. Cui, M. Li, Y. Xu, M. Xu, Y. Du, N. Jiang, F. Huang, C.-T. Lin, Direct formation of wafer-scale single-layer graphene films on the rough surface substrate by PECVD. *Carbon* **129**, 456–461 (2018).

19. Z. Cheng, M. Xia, S. Liu, R. Hu, G. Liang, S. Zhang, Role of rough substrate on the growth of large single-crystal MoS_2 by chemical vapor deposition. *Appl. Surf. Sci.* **476**, 1008–1015 (2019).

20. Z. Hu, M. Xue, Z. Zhang, W. Guo, B. I. Yakobson, Growth instability of 2D materials on non-euclidean surfaces. *ACS Nano* **17**, 12216–12224 (2023).

21. L. Huang, F. Zheng, Q. Deng, Q. H. Thi, L. W. Wong, Y. Cai, N. Wang, C.-S. Lee, S. P. Lau, T. H. Ly, J. Zhao, Anomalous fracture in two-dimensional rhenium disulfide. *Sci. Adv.* **6**, eabc2282 (2020).

22. H. Yang, J. G. Lozano, T. J. Pennycook, L. Jones, P. B. Hirsch, P. D. Nellist, Imaging screw dislocations at atomic resolution by aberration-corrected electron optical sectioning. *Nat. Commun.* **6**, 7266 (2015).

23. H. Sha, Y. Ma, G. Cao, J. Cui, W. Yang, Q. Li, R. Yu, Sub-nanometer-scale mapping of crystal orientation and depth-dependent structure of dislocation cores in $SrTiO_3$. *Nat. Commun.* **14**, 162 (2023).

24. L. W. Wong, K. Yang, W. Han, X. Zheng, H. Y. Wong, C. S. Tsang, C.-S. Lee, S. P. Lau, T. H. Ly, M. Yang, J. Zhao, Deciphering the ultra-high plasticity in metal monochalcogenides. *Nat. Mater.* **23**, 196–204 (2024).

25. Z. Zhang, W. Wang, Z. Dong, X. Yang, F. Liang, X. Chen, C. Wang, C. Luo, J. Zhang, X. Wu, L. Sun, J. Chu, The trends of in situ focused ion beam technology: Toward Preparing transmission electron microscopy lamella and devices at the atomic scale. *Adv. Electron. Mater.* **8**, 2101401 (2022).

26. N. D. Bassim, B. T. De Gregorio, A. L. D. Kilcoyne, K. Scott, T. Chou, S. Wirick, G. Cody, R. M. Stroud, Minimizing damage during FIB sample preparation of soft materials. *J. Microsc.* **245**, 288–301 (2012).

27. J. Mayer, L. A. Giannuzzi, T. Kamino, J. Michael, TEM sample preparation and FIB-induced damage. *MRS Bull.* **32**, 400–407 (2007).

28. J. P. McCaffrey, M. W. Phaneuf, L. D. Madsen, Surface damage formation during ion-beam thinning of samples for transmission electron microscopy. *Ultramicroscopy* **87**, 97–104 (2001).

29. J. Zhou, J. Lin, X. Huang, Y. Zhou, Y. Chen, J. Xia, H. Wang, Y. Xie, H. Yu, J. Lei, D. Wu, F. Liu, Q. Fu, Q. Zeng, C.-H. Hsu, C. Yang, L. Lu, T. Yu, Z. Shen, H. Lin, B. I. Yakobson, Q.

Liu, K. Suenaga, G. Liu, Z. Liu, A library of atomically thin metal chalcogenides. *Nature* **556**, 355–359 (2018).

30. J. N. Coleman, M. Lotya, A. O'Neill, S. D. Bergin, P. J. King, U. Khan, K. Young, A. Gaucher, S. De, R. J. Smith, I. V. Shvets, S. K. Arora, G. Stanton, H.-Y. Kim, K. Lee, G. T. Kim, G. S. Duesberg, T. Hallam, J. J. Boland, J. J. Wang, J. F. Donegan, J. C. Grunlan, G. Moriarty, A. Shmeliov, R. J. Nicholls, J. M. Perkins, E. M. Grieveson, K. Theuwissen, D. W. McComb, P. D. Nellist, V. Nicolosi, Two-dimensional nanosheets produced by liquid exfoliation of layered materials. *Science* **331**, 568–571 (2011).

31. W. S. Yun, S. W. Han, S. C. Hong, I. G. Kim, J. D. Lee, Thickness and strain effects on electronic structures of transition metal dichalcogenides: 2H-MX_2 semiconductors (M = Mo, W; X = S, Se, Te). *Phys. Rev. B* **85**, 033305 (2012).

32. H. Zeng, J. Dai, W. Yao, D. Xiao, X. Cui, Valley polarization in MoS_2 monolayers by optical pumping. *Nat. Nanotechnol.* **7**, 490–493 (2012).

33. Y. Zhao, S. Jin, Stacking and twisting of layered materials enabled by screw dislocations and non-euclidean surfaces. *Acc. Mater. Res.* **3**, 369–378 (2022).

34. P. Wang, Y. Huan, P. Yang, M. Cheng, J. Shi, Y. Zhang, Controlled syntheses and multifunctional applications of two-dimensional metallic transition metal dichalcogenides. *Acc. Mater. Res.* **2**, 751–763 (2021).

35. Y. M. Shi, H. N. Li, L. J. Li, Recent advances in controlled synthesis of two-dimensional transition metal dichalcogenides via vapour deposition techniques. *Chem. Soc. Rev.* **44**, 2744–2756 (2015).

36. J. P. Shi, M. Hong, Z. P. Zhang, Q. Q. Ji, Y. F. Zhang, Physical properties and potential applications of two-dimensional metallic transition metal dichalcogenides. *Coord. Chem. Rev.* **376**, 1–19 (2018).

37. Z. P. Zhang, Y. Gong, X. L. Zou, P. Liu, P. F. Yang, J. P. Shi, L. Y. Zhao, Q. Zhang, L. Gu, Y. F. Zhang, Epitaxial growth of two-dimensional metal-semiconductor transition-metal

dichalcogenide vertical stacks (VSe_2/MX_2) and their band alignments. *ACS Nano* **13**, 885–893 (2019).

38. J. Li, X. Yang, Y. Liu, B. Huang, R. Wu, Z. Zhang, B. Zhao, H. Ma, W. Dang, Z. Wei, K. Wang, Z. Lin, X. Yan, M. Sun, B. Li, X. Pan, J. Luo, G. Zhang, Y. Liu, Y. Huang, X. Duan, X. Duan, General synthesis of two-dimensional van der Waals heterostructure arrays. *Nature* **579**, 368–374 (2020).

39. A. Berkdemir, H. R. Gutiérrez, A. R. Botello-Méndez, N. Perea-López, A. L. Elías, C.-I. Chia, B. Wang, V. H. Crespi, F. López-Urías, J.-C. Charlier, H. Terrones, M. Terrones, Identification of individual and few layers of WS_2 using Raman Spectroscopy. *Sci. Rep.* **3**, 1755 (2013).

40. J. Li, W. Su, F. Chen, L. Fu, S. Ding, K. Song, X. Huang, L. Zhang, Atypical defect-mediated photoluminescence and resonance Raman Spectroscopy of monolayer WS_2. *J. Phys. Chem. C* **123**, 3900–3907 (2019).

41. P. Hajiyev, C. Cong, C. Qiu, T. Yu, Contrast and Raman spectroscopy study of single- and few-layered charge density wave material: 2H-$TaSe_2$. *Sci. Rep.* **3**, 2593 (2013).

42. H. M. Hill, A. F. Rigosi, S. Krylyuk, J. Tian, N. V. Nguyen, A. V. Davydov, D. B. Newell, A. R. Hight Walker, Comprehensive optical characterization of atomically thin $NbSe_2$. *Phys. Rev. B* **98**, 165109 (2018).

43. S. Najmaei, Z. Liu, W. Zhou, X. Zou, G. Shi, S. Lei, B. I. Yakobson, J.-C. Idrobo, P. M. Ajayan, J. Lou, Vapour phase growth and grain boundary structure of molybdenum disulphide atomic layers. *Nat. Mater.* **12**, 754–759 (2013).

44. B. L. Liu, M. Fathi, L. Chen, A. Abbas, Y. Q. Ma, C. W. Zhou, Chemical vapor deposition growth of monolayer WSe_2 with tunable device characteristics and growth mechanism study. *ACS Nano* **9**, 6119–6127 (2015).

45. B. Lyu, J. Chen, S. Wang, S. Lou, P. Shen, J. Xie, L. Qiu, I. Mitchell, C. Li, C. Hu, X. Zhou, K. Watanabe, T. Taniguchi, X. Wang, J. Jia, Q. Liang, G. Chen, T. Li, S. Wang, W. Ouyang,

O. Hod, F. Ding, M. Urbakh, Z. Shi, Graphene nanoribbons grown in hBN stacks for high-performance electronics. *Nature* **628**, 758–764 (2024).

46. D. Mandelli, W. Ouyang, M. Urbakh, O. Hod, The princess and the nanoscale pea: Long-range penetration of surface distortions into layered materials stacks. *ACS Nano* **13**, 7603–7609 (2019).

47. J. W. Martin, J. L. Fogg, G. R. Francas, K. J. Putman, E. P. Turner, I. Suarez-Martinez, N. A. Marks, Graphite rapidly forms via annihilation of screw dislocations. *Carbon* **215**, 118386 (2023).

48. F. Li, H. Nian, K. Du, J. Wang, J. Wang, Y. Zhou, Direct observation of a screw dislocation normal to the beam by Z-Contrast STEM. *J. Am. Ceram. Soc.* **95**, 466–468 (2012).

49. G. Jin, C.-S. Lee, X. Liao, J. Kim, Z. Wang, O. F. N. Okello, B. Park, J. Park, C. Han, H. Heo, J. Kim, S. H. Oh, S.-Y. Choi, H. Park, M.-H. Jo, Atomically thin three-dimensional membranes of van der Waals semiconductors by wafer-scale growth. *Sci. Adv.* **5**, eaaw3180 (2019).

50. J. Li, X. Yang, Z. Zhang, W. Yang, X. Duan, X. Duan, Towards the scalable synthesis of two-dimensional heterostructures and superlattices beyond exfoliation and restacking. *Nat. Mater.* **23**, 1326–1338 (2024).

51. J. Peng, C. Ren, W. Zhang, H. Chen, X. Pan, H. Bai, F. Jing, H. Qiu, H. Liu, Z. Hu, Spatially dependent electronic structures and excitons in a marginally twisted Moiré superlattice of spiral WS_2. *ACS Nano* **16**, 21600–21608 (2022).

52. P. V. Sarma, A. Kayal, C. H. Sharma, M. Thalakulam, J. Mitra, M. M. Shaijumon, Electrocatalysis on edge-rich spiral WS_2 for hydrogen evolution. *ACS Nano* **13**, 10448–10455 (2019).

53. M. Su, W. Zhou, L. Liu, M. Chen, Z. Jiang, X. Luo, Y. Yang, T. Yu, W. Lei, C. Yuan, Micro eddy current facilitated by screwed MoS_2 structure for enhanced hydrogen evolution reaction. *Adv. Funct. Mater.* **32**, 2111067 (2022).

54. X. Fan, Z. Ji, R. Fei, W. Zheng, W. Liu, X. Zhu, S. Chen, L. Yang, H. Liu, A. Pan, R. Agarwal, Mechanism of extreme optical nonlinearities in spiral WS_2 above the bandgap. *Nano Lett.* **20**, 2667–2673 (2020).

55. K. Wang, A. A. Puretzky, Z. Hu, B. R. Srijanto, X. Li, N. Gupta, H. Yu, M. Tian, M. Mahjouri-Samani, X. Gao, A. Oyedele, C. M. Rouleau, G. Eres, B. I. Yakobson, M. Yoon, K. Xiao, D. B. Geohegan, Strain tolerance of two-dimensional crystal growth on curved surfaces. *Sci. Adv.* **5**, eaav4028 (2019).

56. G. Meng, J. Paulose, D. R. Nelson, V. N. Manoharan, Elastic instability of a crystal growing on a curved surface. *Science* **343**, 634–637 (2014).

57. A. R. Bausch, M. J. Bowick, A. Cacciuto, A. D. Dinsmore, M. F. Hsu, D. R. Nelson, M. G. Nikolaides, A. Travesset, D. A. Weitz, Grain boundary scars and spherical crystallography. *Science* **299**, 1716–1718 (2003).

58. H. Yu, N. Gupta, Z. Hu, K. Wang, B. R. Srijanto, K. Xiao, D. B. Geohegan, B. I. Yakobson, Tilt grain boundary topology induced by substrate topography. *ACS Nano* **11**, 8612–8618 (2017).

59. N. P. Mitchell, V. Koning, V. Vitelli, W. T. M. Irvine, Fracture in sheets draped on curved surfaces. *Nat. Mater.* **16**, 89–93 (2017).

60. J. He, K. Hummer, C. Franchini, Stacking effects on the electronic and optical properties of bilayer transition metal dichalcogenides MoS_2, $MoSe_2$, WS_2, and WSe_2. *Phys. Rev. B* **89**, 075409 (2014).

61. Z. L. Hu, P. K. Zhang, W. L. Guo, Z. H. Zhang, B. I. Yakobson, Stress-dominated growth of two-dimensional materials on nonplanar substrates. *J. Mech. Phys. Solids* **157**, 104645 (2021).

62. G. Kresse, J. Furthmüller, Efficiency of ab-initio total energy calculations for metals and semiconductors using a plane-wave basis set. *Comput. Mater. Sci.* **6**, 15–50 (1996).

63. G. Kresse, J. Furthmüller, Efficient iterative schemes for ab initio total-energy calculations using a plane-wave basis set. *Phys. Rev. B* **54**, 11169–11186 (1996).

64. P. E. Blöchl, Projector augmented-wave method. *Phys. Rev. B* **50**, 17953–17979 (1994).

65. J. P. Perdew, K. Burke, M. Ernzerhof, Generalized Gradient Approximation Made Simple. *Phys. Rev. Lett.* **77**, 3865–3868 (1996).

66. R. Nelson, C. Ertural, J. George, V. L. Deringer, G. Hautier, R. Dronskowski, LOBSTER: Local orbital projections, atomic charges, and chemical-bonding analysis from projector-augmented-wave-based density-functional theory. *J. Comput. Chem.* **41**, 1931–1940 (2020).

67. A. Karma, M. Plapp, Spiral surface growth without desorption. *Phys. Rev. Lett.* **81**, 4444–4447 (1998).

68. L. Cai, M. J. Shearer, Y. Zhao, Z. Hu, F. Wang, Y. Zhang, K. W. Eliceiri, R. J. Hamers, W. Yan, S. Wei, M. Tang, S. Jin, Chemically derived kirigami of WSe_2. *J. Am. Ceram. Soc.* **140**, 10980–10987 (2018).

69. E. Meca, J. Lowengrub, H. Kim, C. Mattevi, V. B. Shenoy, Epitaxial graphene growth and shape dynamics on copper: Phase-field modeling and experiments. *Nano Lett.* **13**, 5692–5697 (2013).

70. V. I. Artyukhov, Z. Hu, Z. Zhang, B. I. Yakobson, Topochemistry of Bowtie- and Star-shaped metal dichalcogenide nanoisland formation. *Nano Lett.* **16**, 3696–3702 (2016).

71. I. J. Beyerlein, A. Hunter, Understanding dislocation mechanics at the mesoscale using phase field dislocation dynamics. *Philos. Trans. R. Soc. A* **374**, 20150166 (2016).

72. S. Furukawa, H. Ihara, Y. Murata, Y. Tsukada, T. Koyama, Simulation of dislocation recovery in lath martensite steels using the phase-field method. *Comput. Mater. Sci.* **119**, 108–113 (2016).

73. S. J. Pennycook, D. E. Jesson, High-resolution Z-contrast imaging of crystals. *Ultramicroscopy* **37**, 14–38 (1991).
